# Cancer-associated fibroblast-derived SOD3 enhances lymphangiogenesis to drive metastasis in lung adenocarcinoma

**DOI:** 10.1007/s10456-025-10005-9

**Published:** 2025-09-30

**Authors:** May Wathone Oo, Takao Hikita, Tomoha Mashima, Kosuke Torigata, Yin Min Thu, Tomohiro Habu, Hotaka Kawai, Toshiaki Ohara, Shuta Tomida, Sachio Ito, Ken Suzawa, Hitoshi Nagatsuka, Shinichi Toyooka, Masanori Nakayama

**Affiliations:** 1 Department of Pathophysiology and Drug Discovery, Graduate School of Medicine, Dentistry and Pharmaceutical Sciences, Okayama University, Okayama, Japan; 2 School of Medicine, Kobe University, Kobe, Japan; 3 Department of General Thoracic Surgery and Breast and Endocrinological Surgery, Graduate School of Medicine, Dentistry and Pharmaceutical Sciences, Okayama University, Okayama, Japan; 4 Department of Thoracic Surgery, National Hospital Organization, Shikoku Cancer Center, Matsuyama, Japan; 5 Department of Oral Pathology and Medicine, Graduate School of Medicine, Dentistry and Pharmaceutical Sciences, Okayama University, Okayama, Japan; 6 Department of Pathology and Experimental Medicine, Graduate School of Medicine, Dentistry and Pharmaceutical Sciences, Okayama University, Okayama, Japan; 7 Center for Comprehensive Genomic Medicine, Okayama University Hospital, Okayama, Japan

**Keywords:** Cancer-associated fibroblast, Superoxide dismutase 3, Lymphangiogenesis, Angiogenesis, Metastasis, Lung adenocarcinoma

## Abstract

Despite advancements in diagnostic and therapeutic strategies, lung adenocarcinoma (LUAD) remains a leading cause of cancer-related mortality due to its aggressive metastatic potential. Extracellular superoxide dismutase (SOD3) is an antioxidant enzyme that regulates oxidative stress and is regarded as a tumor suppressor. However, studies have demonstrated that SOD3 can either promote or inhibit cell proliferation and survival in various cancers, and its molecular mechanisms within the tumor microenvironment are poorly understood. In this study, we report a breakthrough in uncovering the role of SOD3 derived from cancer-associated fibroblasts (CAFs) in LUAD. Using LUAD xenograft models co-implanted with SOD3-overexpressing CAFs (CAF^SOD3^), we observe an aggressive tumor phenotype characterized by increased lymphangiogenesis and lymphatic vessel invasion (LVI) of the tumor. Additionally, LUAD patients with elevated SOD3 levels exhibit a higher incidence of LVI and metastasis. Notably, RNA sequencing of CAF^SOD3^ reveals that SOD3-mediated VEGF-dependent tumor progression and lymphangiogenesis are up-regulated. Furthermore, single-cell transcriptomic analysis of LUAD clinical samples confirms a strong correlation between SOD3 expression in fibroblasts and characteristics of tumor exacerbation, such as lymphangiogenesis and metastasis. These findings underscore new insights into the role of CAF-derived SOD3 in LUAD progression and highlight its potential as a biomarker and therapeutic target.

**Electronic supplementary material:**

The online version of this article (10.1007/s10456-025-10005-9) contains supplementary material, which is available to authorized users.

## Introduction

Lung cancer is the leading cause of cancer-related deaths worldwide. Lung adenocarcinoma (LUAD), the most prevalent subtype of non-small cell lung cancer (NSCLC), comprises approximately 40% of NSCLC cases. Despite significant advancements in early diagnosis and treatment methods, a cohort study on metastatic LUAD indicates that patients have a low 5-year survival rate of only 6% [1–3]. In particular, lung cancer with a stroma-rich tumor microenvironment (TME) has been shown to have a poor prognosis and an elevated risk of relapse [4, 5].

While various cell types, including tumor, stromal, and immune cells, contribute to the formation of the TME, cancer-associated fibroblasts (CAFs) serve as primary components in the stroma. Factors secreted by CAFs, such as growth factors, chemokines, cytokines, exosomes, and extracellular matrix (ECM) components, promote capillary formation, lymphangiogenesis, tumor invasion, and metastasis [6]. Hypoxia triggers a wide range of physiological and pathological responses in mammalian cells. In normal tissue, hypoxia stimulates the expression of vascular endothelial growth factor (VEGF), promoting angiogenesis for tissue growth or wound healing [7]. In contrast, intratumoral hypoxia is a hallmark of solid tumors and profoundly influences the malignant phenotype of cancer cells [8] while also inducing an inflammatory phenotype in immune cells and CAFs [9, 10].

Hypoxia-induced reactive oxygen species (ROS) cause the reversible oxidation of cysteine thiol groups, leading to structural modifications that significantly alter protein function [11]. Since the concentration and distribution of ROS determine the pathological effects of the cells in TME, some cancers have developed a balanced redox mechanism [12]. To maintain redox balance, antioxidant enzymes such as superoxide dismutase (SOD) act as scavengers of ROS and play a crucial role in regulating the redox mechanism [13]. Among SOD enzymes, extracellular SOD (EcSOD), encoded by the *SOD3* gene, is abundant in extracellular space, plasma, lung, and liver and is mainly secreted by fibroblasts, macrophages, and endothelial cells [14–16]. Recent studies have identified *SOD3* as a tumor suppressor gene that inhibits tumor development in colorectal, gastric, and pancreatic cancer [17]. Re-expression of SOD3 in tumor-associated endothelial cells induces perivascular nitric oxide accumulation and reduces vessel leakage by up-regulating vascular endothelial cadherin (VE-cadherin) expression, thereby enhancing tumor response to chemotherapy [18]. In contrast, some studies have reported that high SOD3 expression is associated with a poor survival rate in lung cancer [19]. These facts indicate that the relationship between SOD3 and cancer remains controversial.

Here, we discovered that SOD3 is predominantly expressed in CAFs, and its expression is associated with recurrence in LUAD patients. Using a mouse xenograft LUAD model transplanted with human-derived CAF, we showed that overexpression of SOD3 in CAF induces lymphangiogenesis, resulting in tumor metastasis in the lymph nodes. Mechanistically, transcriptome analysis revealed that SOD3 overexpression in CAF enhanced cancer exacerbation-related genes, particularly angiogenesis, lymph vessel development, collagen degradation, and epithelial-mesenchymal transition. Our findings suggest that SOD3 may be a prognostic factor for LUAD patients, and SOD3-expressing CAF could be a potential therapeutic target to disrupt the stroma-tumor alliance relation in tumor development and progression.

## Materials and methods

### Human samples

Surgical tissue samples (*n* = 56) from patients who underwent surgery for LUAD at Okayama University Hospital were obtained from the surgical pathology database of Okayama University (Okayama, Japan). Tissue sections (Sects) (3–5 μm thick) were prepared for SOD3 expression analysis. This study was conducted in accordance with the ethical principles outlined in the Ethical Review Boards of Okayama University (Approval Number: Ken 2311-043). Appropriate informed consents were obtained from all participants before sample collection and data usage. To ensure strict protection of patient confidentiality, all clinical samples and associated data were de-identified and were assigned a unique, non-identifiable study code. All links to personally identifiable information were securely stored in a password-protected database, ensuring accessibility only to authorized persons. Direct patient identities were not used during the data processing, statistical analysis, or in any part of the published work. Additionally, all clinical samples were handled and stored following institutional biobanking and data management policies to secure ethical and responsible use in research.

### Cell lines and mice

The human NSCLC cell line (PC9), acquired from the RIKEN BRC cell bank (RCB4455), was utilized in this study. Cells were cultured in Dulbecco’s Modified Eagle Medium (DMEM) (043-30085, Fujifilm, Japan) with 10% fetal bovine serum (FBS) (A5256701, Thermo Fisher Scientific, MA, USA) at 37 °C in a humidified atmosphere containing 5% CO_2_. Human umbilical vein endothelial cells (HUVECs), purchased from Lonza (C2519A), were maintained in Endothelial Growth Medium-2 (EGM-2; Lonza, CC-3162) supplemented with the EGM-2 BulletKit (Lonza, CC-4176), which includes endothelial basal medium (EBM-2) and the following growth supplements: 2% FBS, human epidermal growth factor (hEGF), vascular endothelial growth factor (VEGF), fibroblast growth factor-B (hFGF-B), insulin-like growth factor-1 (IGF-1), ascorbic acid, hydrocortisone, and heparin. HUVECs were cultured in tissue culture dishes coated with 2% gelatin at 37 °C in a humidified atmosphere of 5% CO₂. This study employed human LUAD-derived CAFs isolated from patients with pathologically diagnosed LUAD [20]. CAFs were maintained in Roswell Park Memorial Institute (RPMI)-1640 (189–02025, Fujifilm, Japan) medium with 10% FBS (A5256701, Thermo Fisher Scientific, MA, USA) at 37 °C in a humidified incubator with 5% CO_2_. Male nude mice (BALB/c-nu/nu) purchased from Shimizu Laboratory Suppliers were kept under pathogen-free conditions.

### SOD3 overexpression in CAFs and PC9

For SOD3 overexpression in CAFs and PC9, the lentiviral vector pLV[Exp]-mCherry-EF1A > hSOD3 [NM_003102.4] and pLV[Exp]-mCherry/Puro-EF1A > ORF_Stuffer were purchased from Vector Builder. The plasmids were maintained in Escherichia coli and purified using a standard plasmid midi-prep kit (740410.50, NucleoBond^®^ Xtra Midi, Macherey-Nagel, Germany). Virus production was conducted in Lenti-X 293T cells (632180, Takara Bio Inc) utilizing lentiviral packaging plasmids (pVSVG and psPAX2). Lenti-X 293T cells were cultured at a density of 3.0 × 10^5^ cells per well in six-well plates and transfected with lentiviral vectors and packaging plasmids using FuGENE HD transfection reagent (E2311, Promega, Madison, WI). The supernatant viral media was collected 48 h post-transfection and added to CAFs. Single-cell cloning was performed using serial dilution after obtaining the successfully transfected CAF cell pool. Each single cell clone was checked for SOD3 overexpression and mCherry expression, and successful clones were selected to establish the SOD3 overexpression CAF and PC9 cell line (CAF^SOD3^, PC9^SOD3^) and the empty vector transduced CAF cell line (CAF^mCherry^). Successful transfection was identified by the expression of mCherry under a digital light microscope (Keyence, BZ-X800 microscope, Japan), and SOD3 protein expression was evaluated by western blotting. The established CAF^SOD3^ and CAF^mCherry^ cell clones were cultured in RPMI-1640 medium (189–02025, Fujifilm, Japan) with 10% FBS (A5256701, Thermo Fisher Scientific, MA, USA) and PC9^SOD3^ are cultured in DMEM (043-30085, Fujifilm, Japan) at 37 °C in a humidified incubator with 5% CO_2_.

### Single-cell RNA sequence data analysis in LUAD clinical tissue samples

Accession number GSE131907 [21] identified a single-cell RNA sequencing dataset of 208,506 single-cell mRNA expression profiles from 44 patients who were pathologically diagnosed with LUAD (NCBI Gene Expression Omnibus database). Among them, 7 samples are patient-matched normal lung tissues, and the remaining 37 LUAD samples were analyzed using the Seurat package (V5.1.0) in this study [22]. In brief, raw UMI count matrices from each sample and their associated metadata were merged and normalized using the SCTransform function. Clustering and UMAP visualization were then performed. The validity of data integration was evaluated by assessing the consistency of major clusters and cell type annotations provided in the original paper. To further confirm the classification of each cell type, module scores were calculated for lineage marker genes depicted in the original study using the AddModuleScore_UCell function from the UCell package (Supplementary Fig. 1A). To classify 18 advanced LUAD samples (advanced stage lung cancer, brain, and lymph node metastasis), based on SOD3 expression levels in fibroblasts, the median values of the fibroblast population in each sample were used as a threshold to classify samples into SOD3-high and SOD3-low groups. The pseudo-bulk expression matrices of the fibroblast population for each class were obtained using the AggregateExpression function. A ranked gene list was then generated based on statistical metrics using the Find Markersfunction. In total, 15,485 genes were ranked by − log10(p-value) × effect size, where the effect size was defined as 1 for genes with a positive fold change and − 1 for those with a negative fold change. Gene set enrichment analysis (GSEA) was performed in preranked mode. Additionally, Gene Ontology (GO) analysis for Biological Processes and Ingenuity Pathway Analysis (IPA) were performed on genes up-regulated in SOD3-high fibroblasts compared to SOD3-low fibroblasts from advanced-stage LUAD patients. Differentially expressed genes (DEGs) (fold change > ± 1.2, *p* < 0.01) were analysed. The top 20 enriched GO terms were visualized as bubble plots using R (version 4.4.1). To gain an overview of the associated molecules and cellular functions, DEGs were imported into the Ingenuity Pathway Analysis (IPA) software and subjected to Core Analysis using default parameters. A self-generated molecular network was extracted using the Graphical Summary tool.

### Bulk RNA sequence and transcriptomic analysis

CAF and CAF^SOD3^ cells were seeded and cultured until they reached 80–90% confluence. According to the manufacturer’s instructions, the total RNA was isolated and purified using the Monarch Total RNA Miniprep Kit (T2010S, New England Biolabs). The RNA Integrity Number (RIN) of the samples was assessed with the Bioanalyzer (Agilent Technologies). Using the NEBNext^®^ Poly(A) mRNA Magnetic Isolation Module (E7490), mRNA with a poly-A tail was selected and captured using magnetic beads. RNA sequencing libraries were prepared using MGIEasy Fast RNA Prep Set. The size distribution and concentration of the library were checked using the Bioanalyzer (Agilent Technologies) and Qubit (Thermo Fisher Scientific). Sequencing was then performed using DNBSEQ-T7RS High Throughput Sequencing Reagent Kit (V3.0). All sequence reads were converted to FASTQ format using bcl2fastq software. Reads mapping was performed using the RNA-seq pipeline of Illumina’s DRAGEN program (V3.9). The human genome (hg38) was used as the reference sequence (Genome assembly GRCh38.p11), and gencode.v42.chr_patch_hapl_scaff.annotation.gtf was used for the annotation. The raw count data were normalized using the DEseq2 package, and the data were presented as gene counts.

GSEA was conducted on a total gene of 20,829 genes using GSEA (v4.3.2) software downloaded from the GSEA website (http://software.broadinstitute.org/gsea/index.jsp). The gene set was obtained from the Molecular Signature Database (MSigDB) (2024.1). GSEA data were presented as bubble plots generated by R (4.4.1) and enrichment plots. To investigate the effect of CAF in TME, we performed an analysis of secretomes. We observed 1891 genes annotated as secreted in The Human Protein Atlas, and 121 of them were up-regulated in CAF^SOD3^ (121 genes). Then, we found the shared secretome genes associated with the enriched angiogenesis, lymphangiogenesis, and EMT using Venny 2.1. The shared gene expression was presented as a heatmap.

### LUAD xenograft mouse model

For xenograft tumor models, tumors were implanted subcutaneously through co-injection of PC9 cells and CAF, CAF^mCherry^, or CAF^SOD3^. A PC9-only xenograft served as a control. A monolayer of cancer cells and CAFs was harvested by trypsinization, washed, and suspended in PBS at a concentration of 5 × 10^5^ cells per 0.1 ml. Tumor transplantation was performed on fully anesthetized mice using isoflurane inhalation. For tumor cell-only xenografts, PC9 cells (5 × 10^5^) were injected subcutaneously into the flanks of the mice. For co-xenografts, PC9 cells and CAF, CAF^mCherry^, or CAF^SOD3^ were implanted in a 1:1 ratio. After four weeks of tumor growth, mice were euthanized by cervical dislocation, and the specimens were collected for further analysis.

### Tissue processing for histological examination

The harvested mouse tumor and lymph node samples were processed into formalin-fixed paraffin-embedded (FFPE) sections. The samples were fixed in 4% paraformaldehyde for 48 h and subsequently dehydrated in 70, 80, 90, and 100% ethanol and xylene. Finally, the samples were embedded in paraffin, and serial Sect. (4 μm thick) were prepared for hematoxylin and eosin (HE), immunohistochemistry (IHC), and fluorescent IHC.

### Immunohistochemistry

For immunostaining, the following antibodies were used: SOD3 (1:100, AF3420, R&D), CD31 (1:100, 3528, Cell Signaling Technology), α-SMA (1:100, 19245, Cell Signaling Technology), LYVE1 (1:50, MAB2125, R&D), CK7 (1:50, 4898, Cell Signaling Technology), CK7 (1:40, M7018, Dako), and Podoplanin (1:50, M3619, Dako). All FFPE sections were deparaffinized and rehydrated, followed by H_2_O_2_ quenching. Subsequently, relevant antigen retrieval was performed, and sections were incubated with a normal horse-blocking agent for 20 min at room temperature. The primary antibodies were incubated overnight at 4 °C. The secondary antibodies were applied using an Impress kit (Vector Laboratories, USA). Signal enhancement was achieved with 3, 3’-diaminobenzidine (DAB) (11009-41, Histofine DAB substrate), and nuclear counterstaining with hematoxylin was performed. After dehydration through a graded series of ethanol followed by xylene, the specimens were mounted using New M/X mounting medium (Tokyo Glass Instruments). The staining results were evaluated using an optical microscope (Olympus, BX43, Japan).

### Fluorescent immunohistochemistry

Fluorescent immunohistochemistry for SOD3, α-SMA, and CK7 was performed. After the tissue sections were deparaffinized and rehydrated, antigen retrieval was conducted using microwave heating in 0.01 M sodium citrate buffer (pH 6.0) for 5 min. Next, the sections were incubated in a blocking solution containing 1% BSA, 2% normal donkey serum, and 0.1% Tween 20 in Tris-buffered saline (TBS) (0.05 M Tris base, 0.155 M NaCl) for 20 min at room temperature. The sections were then incubated with primary antibodies overnight at 4 °C. The primary antibodies used were SOD3 (1:100, AF3420, R&D), α-SMA (1:200, 19245, Cell Signaling Technology), and CK7 (1:40, M7018, Dako). Secondary antibodies were diluted to 1:100 and applied for 1 h at room temperature. The secondary antibodies used included Alexa Fluor 555 anti-rabbit IgG (A32794, Thermo Fisher Scientific, MA, USA) for α-SMA, Alexa Fluor 488 anti-goat IgG (A32814, Thermo Fisher Scientific, MA, USA) for SOD3, and Alexa Fluor 647 anti-rat IgG (A21247, Life Science) for CK7. After the reaction, the sections were stained with Hoechst 33342 (Dojindo Molecular Technologies) and mounted with Fluoromount-G (00495802, Thermo Fisher Scientific, MA, USA). The staining results were observed using a ZEISS LSM 780 confocal microscope (Germany).

### Western blotting

Cells were washed with cold PBS, and whole cell lysate was extracted with a lysis buffer, a mixture of RIPA buffer (25 mM Tris-HCl (pH 7.6), 150 mM NaCl, 1% NP-40, 1% sodium deoxycholate, 0.1% SDS), a complete mini protease inhibitor cocktail (Roche, Basel, Switzerland), and phosphate inhibitor cocktails 2 and 3 (Sigma-Aldrich, Burlington, MA, USA). After normalization of protein concentration, samples were mixed with 3×SDS sample buffer (188 mM Tris-HCl (pH 6.8), 3% SDS, 30% Glycerol, 0.01% Bromophenol blue) and boiled at 95 °C for 10 min. Protein samples were separated by sodium dodecyl sulfate-polyacrylamide gel electrophoresis (SDS-PAGE) and transferred to polyvinylidene difluoride (PVDF) membranes. The membranes were blocked in 3% skim milk (31149-75, Nacalai Tesque, Japan) prepared in TBS-T (0.05 M Tris base, 0.155 M NaCl, 0.05% Tween 20) for 1 h with shaking at room temperature. The primary antibodies were applied to the membrane and incubated overnight at 4 °C. The primary antibodies for western blot analysis were SOD3 (1:1000, AF3420, R&D), VEGFA (1:1000, 50661 CST), VEGFR2 (1:1000, 2479, CST), p-VEGFR2 (1:1000, 2478, CST), E-cadherin (1:1000, 13-1900, Thermo Fisher Scientific, MA, USA), N-cadherin (1:1000, 13116, Cell Signaling Technology), and β-actin (1:1000, 81115-1-RR, Proteintech). Then, the membranes were incubated with horseradish peroxidase (HRP) conjugated secondary antibodies for 1 h with shaking at room temperature. Before and after secondary antibody incubation, 10 min, three times washes with TBS-T with shaking were performed. Blots were visualized with Fusion Solo 7 S Imaging System (Vilber, France).

### Quantitative reverse transcription PCR (qRT-PCR)

The total RNA was isolated and purified using the Monarch Total RNA Miniprep Kit (T2010S, New England Biolabs). The quantity of RNA was assessed using an Implen NanoPhotometer (Implen GmbH). For cDNA synthesis, 500 ng to 1 µg of total RNA was reverse-transcribed using the SuperScript IV VILO Master Mix (11756050, Thermo Fisher Scientific, MA, USA) in a total reaction volume of 20 µL. qRT-PCR was performed using the KAPA SYBR FAST qPCR Master Mix (2×) Kit (KAPA Biosystems) on a Bio-Rad real-time PCR system. Each reaction was carried out in a total volume of 20 µL containing 1 µL of cDNA template, 10 µL of 2× master mix, and 0.4 µM of each primer. The thermal cycling conditions were as follows: initial denaturation at 95 °C for 30 s, followed by 40 cycles of 95 °C for 5 s and 60 °C for 30 s.

Gene expression levels were normalized to the housekeeping gene ACTB (β-Actin) or PRL13A and calculated using the 2^−ΔΔCt^ method. Primer sequences used for amplification are listed in Supplementary Table 1.

### DCFDA

Intracellular ROS levels were assessed using the DCFDA Cellular ROS Detection Assay Kit (Abcam, ab113851), following the manufacturer’s instructions. Briefly, cells (2.5 × 10^4^) were seeded into 96-well plates (Black, clear bottom) and allowed to attach overnight. The next day, cells were washed with 1X buffer provided in the kit and incubated with 25 µM DCFDA solution at 37 °C for 45 min in the dark. After incubation, cells were washed, and 1X buffer was added. Fluorescence intensity was measured immediately using a FlexStation 3 Multi-Mode Microplate Reader (Molecular Devices) (excitation: 485 nm, emission: 535 nm). ROS levels were expressed as relative fluorescence intensity normalized to control groups. All experiments were performed in triplicate, and results are shown as mean ± SD.

### Treatment of PC9 cells with conditioned medium

We prepared different conditioned media (CM) from CAF^mCherry^, or CAF^SOD3^ cells. Cells (5 × 10^5^) were plated to 6-well plates for 72 h. The cultured media were collected and centrifuged at 1000×g for 10 min to remove the cell debris. Then, the supernatant was collected and sterile-filtered at 0.22 μm. Before treatment with CM, PC9 cells were seeded and cultured to obtain 80% confluency. Culture media were replaced with the CM (CAF^mCherry^-CM, or CAF^SOD3^-CM) and cultured for 48 h. After treatment, proteins were extracted for the western blotting.

### VEGFR phosphorylation assay

To assess the activation of VEGF signaling, VEGFR2 phosphorylation was examined by western blotting. HUVECs were serum-starved in EGM-2 basal medium (without supplements) for 4 h to reduce baseline phosphorylation levels. After starvation, cells were stimulated for 10 min at 37 °C with different conditioned media (CAF-CM, CAF^mCherry^-CM, or CAF^SOD3^-CM). As a positive and negative control, EGM-2 containing 50 ng/mL human recombinant VEGF-165 (48143, Cell Signaling Technology), and EGM-2 without VEGF, were used, respectively. After stimulation, cells were immediately placed on ice, washed with cold PBS, and lysed in RIPA buffer supplemented with protease and phosphatase inhibitors. Protein concentrations were normalised, and western blotting was performed to observe total VEGFR2 and phosphorylated VEGFR2.

### HUVECs tube formation assay

For tube formation, 96-well plates were pre-coated with 50 µL of Matrigel (Corning, 356231) and incubated at 37 °C for 30 min to allow gel polymerization. HUVECs were harvested and resuspended in conditioned medium (CAF-CM, CAF^mCherry^-CM, or CAF^SOD3^-CM) and seeded onto the Matrigel-coated wells at a density of 1 × 10⁴ cells/well. As a positive and negative control, EGM-2 containing 50 ng/mL human recombinant VEGF-165 (48143, Cell Signaling Technology), and EGM-2 without VEGF, were used, respectively. After 24 h of incubation at 37 °C in a 5% CO₂ incubator, tube-like structures were visualized using an Evident microscope (APX100). Images were captured at 10× magnification and analyzed using ImageJ (v1.52a).

### HUVECs transwell migration assay

HUVECs migration was assessed using 24-well transwell inserts with 8.0 μm pore size polycarbonate membranes (Falcon, 353097). Inserts were placed into wells containing 600 µL of EGM-2 medium with or without VEGF (50ng/ml), and conditioned medium (CAF-CM, CAF^mCherry^-CM, or CAF^SOD3^-CM). HUVECs were suspended in EGM-2 and seeded into the upper chamber at a density of 1 × 10^5^ cells per insert in 200 µL. After 24 h of incubation at 37 °C, non-migrated cells on the upper membrane surface were gently removed with a cotton swab. Migrated cells on the lower surface were fixed and stained with Diff Quick solution (Nanjing Jiancheng Bioengineering Institute) according to the manufacturer’s instructions. After drying up, the membrane was carefully cut and mounted with Fluoromount-G (00495802, Thermo Fisher Scientific, MA, USA) on the glass slide. Stained cells were imaged using an Evident microscope (APX100). Images were captured at 20× magnification and analyzed using ImageJ (v1.52a).

### Analysis of SOD3 expression in LUAD patient samples and correlation with clinical outcomes

To evaluate SOD3 expression in LUAD tissues, IHC staining was performed on FFPE tumor samples. SOD3 protein expression levels were semi-quantitatively assessed by two independent observers using a standard staining intensity scoring system ranging from 0 to 3: 0 (no staining), 1 (weak), 2 (moderate), and 3 (strong) (Supplementary Fig. 1B). Based on these scores, samples were stratified into SOD3-low (Score 0, 1) and SOD3-high (Score 2, 3) groups for further analysis. The association between SOD3 expression and clinicopathological parameters, including recurrence status, was assessed and summarized in Table 1. Statistical analyses were performed using Chi-square or Fisher’s exact test, as appropriate.

**Table 1:** Association between SOD3 expression and clinicopathological characteristics of patients with LUAD

		SOD3 Expression		
Characteristics	Total LUAD *n* = 56	High n (% of total)	Low n (% of total)	*P*-value
Age, years				0.0434*
< 60	7	4 (57.14)	3 (42.86)	
≥ 60	49	21 (42.86)	28 (57.14)	
Gender				0.3358
Male	29	12 (41.38)	17 (58.62)	
Female	27	13 (48.15)	14 (51.85)	
Smoking				0.9565
Never	27	12 (44.44)	15 (55.56)	
Ever	29	13 (44.83)	16 (55.17)	
pT classification				< 0.0001*
T1	26	9 (34.62)	17 (65.38)	
T2	29	15 (51.72)	14 (48.28)	
T3/4	1	1 (100)	0 (0)	
Lymph node metastasis				< 0.0001*
No	52	21 (40.38)	31 (59.62)	
Yes	4	4 (100)	0 (0)	
LVI				< 0.0001*
No	33	6 (18.18)	27 (81.82)	
Yes	23	19 (82.61)	4 (17.39)	
Recurrence				< 0.0001*
No	45	17 (37.78)	28 (62.22)	
Yes	11	8 (72.73)	3 (27.27)	

LUAD, Lung adenocarcinoma; pT, Pathological T stage; n, Number of patients. Ever, Smoking at any time from the beginning of life

*P*-value, the difference in clinicopathological characteristics between the SOD3 high and low expression groups. *P < 0.05 was considered statistically significant. Fisher’s exact test analyzed significance

The prognostic relevance of SOD3 mRNA expression was evaluated using the Kaplan–Meier Plotter (http://kmplot.com; RRID: SCR_024521), an online database that integrates gene expression and clinical outcome data. We analyzed overall survival (OS) in 1161 LUAD patients from The Cancer Genome Atlas (TCGA) dataset. Patients were divided into SOD3-high (*n* = 579) and SOD3-low (*n* = 582) groups based on the median SOD3 mRNA expression (cutoff value = 347). Survival curves were generated using the Kaplan–Meier method, and log-rank tests were performed to evaluate statistical significance. Hazard ratios (HRs) and 95% confidence intervals (CIs) were also calculated.

### Quantification and statistical analysis

IHC staining data quantification was performed on five randomly captured images at high magnification. Counting was conducted using ImageJ (v1.52a). Statistical analysis was carried out with GraphPad Prism 9.1.1. A Student’s t-test for independent samples with equal variances compared two groups, while One-Way ANOVA followed by Tukey’s multiple comparison post hoc test was used to assess differences among more than two groups when necessary. Significant differences were considered at *P* < 0.05.

Tumor size was measured by recording length and width every three days, and calculating tumor volume by using the formula of ½ (length × width^2^), and growth curves were plotted over time. To assess differences in tumor growth kinetics between groups, a two-way repeated measures ANOVA was performed, followed by Tukey’s multiple comparison post hoc test to evaluate statistical significance at individual time points. Data are presented as mean ± standard deviation (SD). A *P* < 0.05 was considered statistically significant.

### Study approval

All animal experiments were conducted according to the protocol established by Okayama University’s Care and Use of Laboratory Animals and approved by the Committee on Ethics of Animal Experiments at Okayama University Graduate School of Medicine, Dentistry, and Pharmaceutical Sciences (OKU-2023661).

## Results

### SOD3 expression in fibroblasts positively correlates with the recurrence in LUAD

**Fig. 1:**
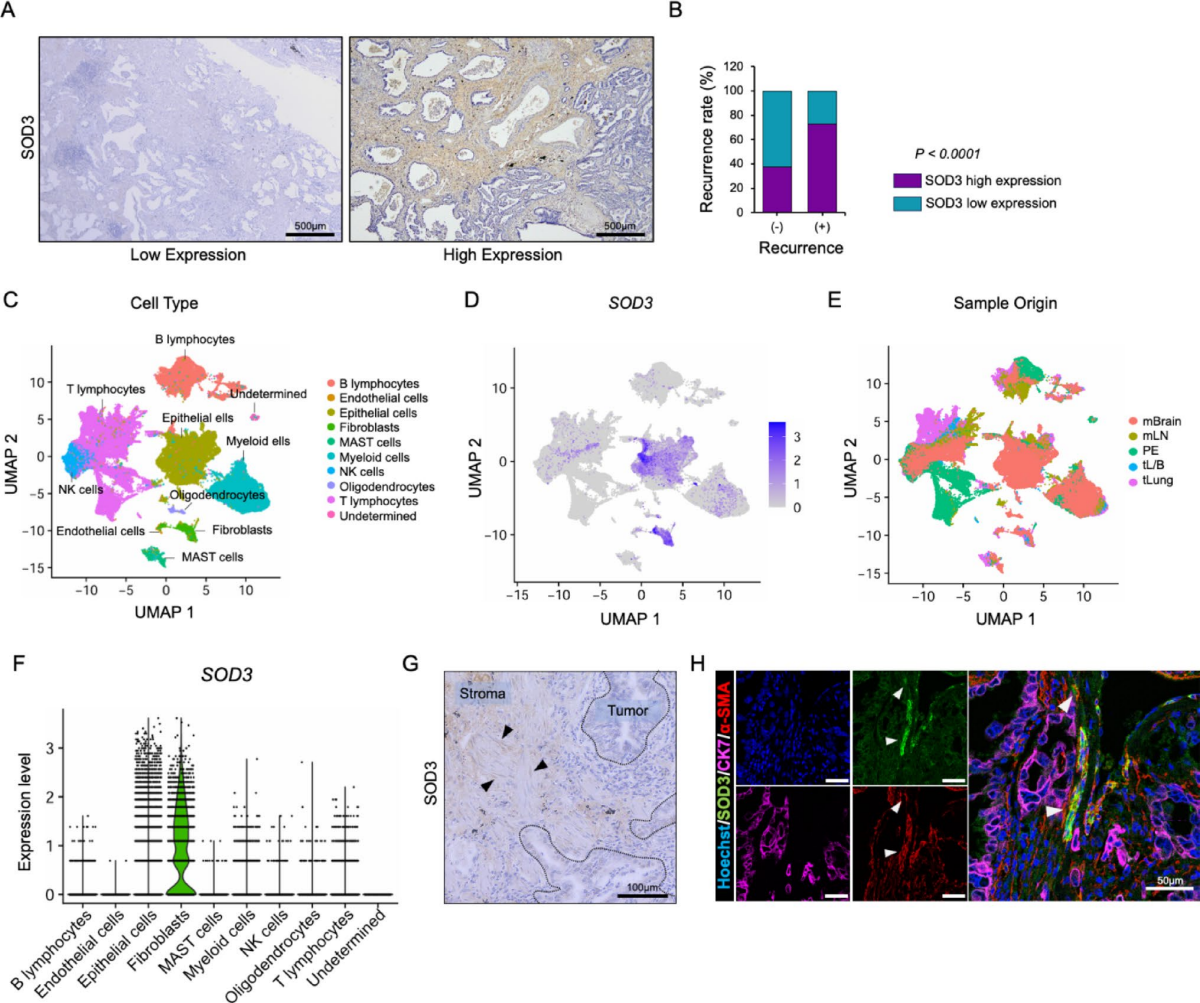
SOD3 expression in fibroblasts positively correlates with recurrence in LUAD. **A** Representative image of SOD3 expression in LUAD clinical samples. **B** Correlation analysis between SOD3 expression and recurrence rate in LUAD clinical samples (*n* = 56). Statistical analysis was performed using Fisher’s exact test, with *P* < 0.05 considered significant. **C** Uniform Manifold Approximation and Projection (UMAP) plot of 208,506 single cells from LUAD clinical samples (GSE131907), color-coded by major cell lineages. **D** UMAP plot showing SOD3 gene expression across major cell types. **E** UMAP displaying the distribution of sample origins across major cell types. mBrain, brain metastases; mLN, lymph nodemetastases; PE, pleural effusion; tL/B, advanced-stage tumor lung; tLung, early-stage tumor lung. **F** Violin plot indicating SOD3 expression level in major cell types. **G** A representative image of the immunohistochemistry of SOD3 in a LUAD clinical sample showing SOD3-positive fibroblasts. Arrowheads show SOD3-positive fibroblasts. **H** A representative image of immunofluorescent staining of SOD3 (green), α-SMA (red), CK7 (magenta), and Hoechst (blue) in a LUAD clinical sample. Arrowheads show the colocalization of SOD3 and α-SMA

To assess the SOD3 expression pattern in LUAD, we performed immunohistochemistry in clinical samples. As shown in Fig. 1A, SOD3 expression varied among patients, demonstrating low and high expression levels. We identified the low and high SOD3 expression by using the standard IHC scoring method, ranging from 0 to 3 (Supplementary Fig. 1B). Detailed information on the patient samples is shown in Table 1. Furthermore, analysis of the association between SOD3 expression and recurrence rates revealed a positive correlation between SOD3 protein levels and LUAD recurrence (Fig. 1B). In addition to tissue-based data analyses, we evaluated overall survival OS in 1161 lung adenocarcinoma (LUAD) patients from the TCGA dataset using the Kaplan-Meier method. The data revealed that high SOD3 expression was significantly associated with poorer OS compared to those with low expression (HR = 1.36, 95% CI = 1.15–1.62, *p* = 0.00039) (Supplementary Fig. 1C).

To identify the major source cells secreting SOD3 in the LUAD TME, we performed a scRNA sequence analysis using a publicly available database by Kim et al. (GSE131907) [21]. Our study revealed that SOD3 was predominantly expressed by fibroblasts, followed by epithelial cells, myeloid cells, and lymphocytes (Fig. 1C-F). IHC on LUAD patient samples further confirmed SOD3 expression in fibroblasts (Fig. 1G). Given the fact that the majority of the fibroblasts in the samples are CAFs, we examined SOD3 expression in CAFs using multiplex immunofluorescent staining for SOD3, α-SMA (CAF marker), and CK7 (lung epithelial cell marker). The colocalization of SOD3 and α-SMA suggests that CAFs in LUAD were the major source of SOD3 (Fig. 1H) in LUAD TME.

### CAF-derived SOD3 alters the phenotype of LUAD cancer cells in vivo

**Fig. 2:**
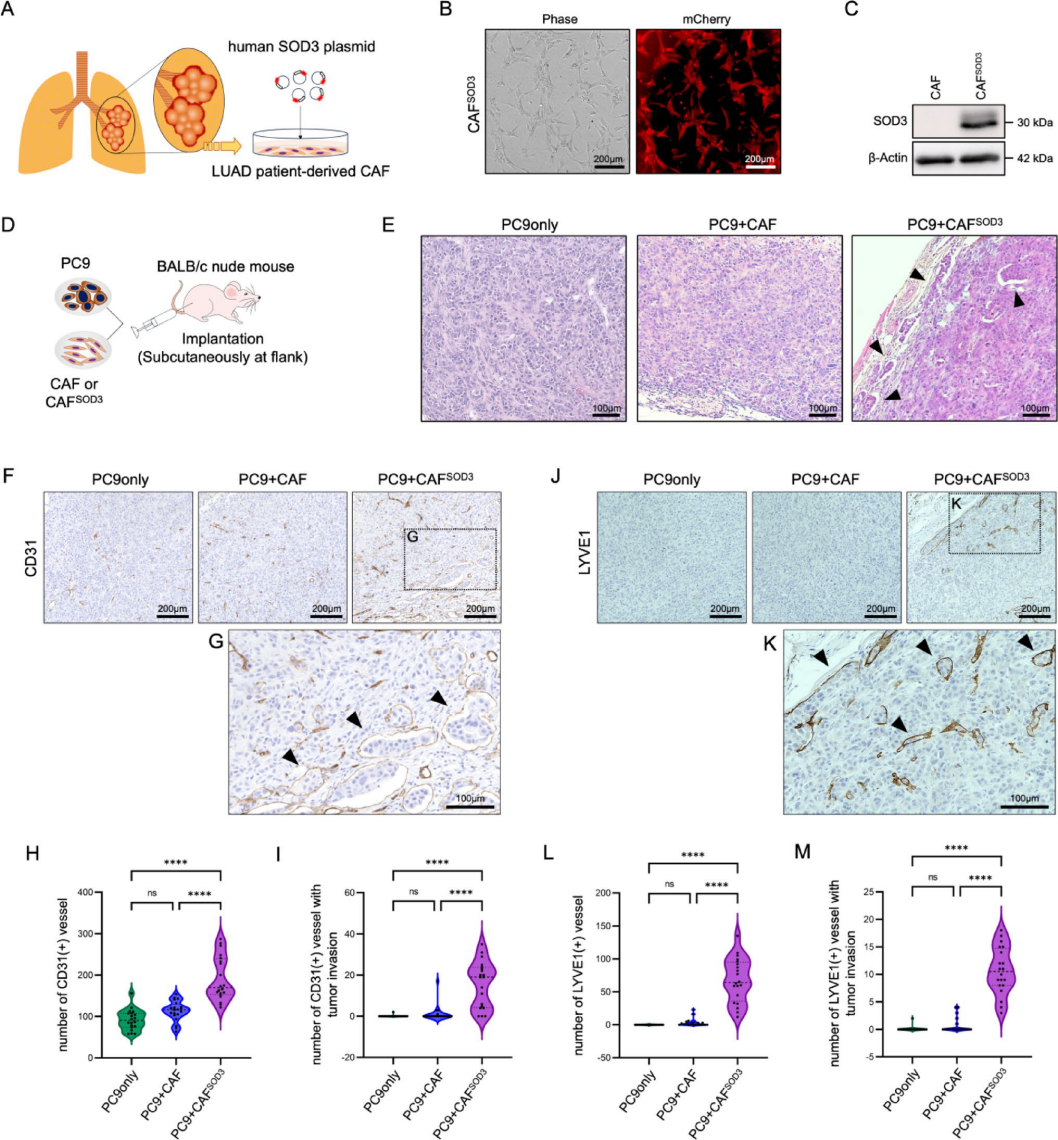
SOD3-rich CAFs’ impact on the tumor phenotype in LUAD xenografts. **A** Schematic illustration of human SOD3 (hSOD3) overexpression in LUAD patient-derived CAFs. The hSOD3 plasmid tagged with mCherry was transfected using the lentiviral gene expression system. A stable SOD3-overexpressing CAF clone (CAF^SOD3^) ) was established via single-cell cloning. **B**. Representative image of CAF**)**^SOD3^. Stable expression was confirmed by mCherry expression under a fluorescent microscope. **C** Confirmation of SOD3 protein expression in established CAF^SOD3^ by immunoblot. **D** Schematic representation of the LUAD xenograft mouse model. Human LUAD cancer cell line (PC9) was co-implanted subcutaneously with CAFs with or without SOD3 overexpression, into the flanks of BALB/c nude mice. **E** HE staining of tumor xenografts. Arrowheads indicate the invasion of tumor cells into split-like structures. **F** Representative images of IHC of CD31 in tumor samples. **G** High-magnification image of CD31 IHC staining of tumor sample implanted with PC9 and CAF^SOD3^ (corresponding to the islet in **F**). **H**,** I** Quantification of CD31-positive blood vessels and CD31-positive blood vessels with tumor invasion. **J** Representative IHC images of LYVE1 in tumor samples. **K** High-magnification image of LYVE1 IHC staining of tumor sample implanted with PC9 and CAF^SOD3^ (corresponding to the islet in **J**). **L**,** M** Quantification of LYVE1-positive lymphatic vessels and LYVE1-positive lymphatic vessels with tumor invasion. CAF, cancer-associated fibroblast extracted from a LUAD patient; CAF^SOD3^, SOD3 overexpression in CAF; PC9 only, PC9 only implanted xenograft; PC9 + CAF, xenograft model with PC9 and CAF; PC9 + CAF^SOD3^, xenograft model with PC9 and CAF^SOD3^. *n* = 4, 5 images with high magnification per sample. All data were presented as the number of signals per image. Statistical analyses were performed using one-way ANOVA followed by Tukey’s multiple-comparison post hoc test; no significant (ns), ****P < 0.0001

To investigate the role of CAF-derived SOD3 in the LUAD TME, we overexpressed SOD3 in LUAD patient-derived CAFs [20]. As shown in Fig. 2A, we transfected isolated CAFs with a human-SOD3 plasmid, which expresses mCherry. The overexpression procedure involved viral transfection, single-cell cloning, and expansion. SOD3 overexpression was confirmed with mCherry fluorescent expression under the microscope and in cell lysates by western blotting (Fig. 2B and C).

To further investigate the influence of SOD3-rich CAFs on tumor development, we established tumor models implanting LUAD cancer cells (PC9) together with either the CAF or the CAF^SOD3^. As shown in Fig. 2D, we co-implanted the tumor cells and CAFs subcutaneously into the flank and measured tumor size by recording length and width every three days (Supplementary Fig. 2A), and calculating tumor volume by using the formula of ½ (length × width^2^). A PC9-only implantation model served as a control for comparison. Tumors in the PC9 co-transplanted with CAF mice exhibited the largest size, followed by the PC9 co-transplanted with CAF^SOD3^ mice and PC9 only transplanted mice. Interestingly, SOD3-overexpressing groups showed significantly higher tumor volume compared to normal CAF transplanted groups shortly after implantation (day 3, 6, and 9 after transplantation). However, no significant difference was observed between the SOD3 overexpressing and non-overexpressing CAF groups over time. (Supplementary Fig. 2B–D). These data suggest that while SOD3 may tend to promote tumor growth, its primary role could be initiating LUAD tumor development rather than sustaining continuous tumor growth. Here, the initial validation comparing CAF and CAF^mCherry^ revealed negligible phenotypic differences (Supplementary Fig. 2E–I). The empty vector did not induce SOD3 expression and did not affect intracellular ROS activity in CAFs (Supplementary Fig. 4E–G). Furthermore, a preliminary xenograft model was used to evaluate the impact of CAF and CAF^mCherry^ on tumor progression. Tumors were allowed to grow until reaching a volume of 1000mm^3^, at which point mice were sacrificed (Supplementary Fig. 2H). No significant difference in tumor growth over time between the PC9 + CAF and PC9 + CAF^mCherry^ groups (Supplementary Fig. 2I).

After 4 weeks of tumor development, tumor samples were harvested. Histological analysis of tumor samples revealed that CAF^SOD3^ injection led to phenotypic changes in tumor cells. While all groups exhibited the malignant characteristics of tumor cells with relatively sparse stromal content, only the tumor cells implanted with CAF^SOD3^ displayed invasive features, particularly tumor cell infiltration into a vessel-like structure (Fig. 2E).

To gain further insight into tumor infiltration into the vessel-like structures, we performed IHC staining using CD31, a blood vessel endothelial cell marker (Fig. 2F and G), and LYVE1, a lymphatic endothelial cell marker (Fig. 2J and K). The quantification results showed a significant increase in CD31-positive and LYVE1-positive vessel-like structures in the CAF^SOD3^ group compared to other groups (Fig. 2H and L). Additionally, tumor cell intravasation was predominantly observed in CAF^SOD3^-injected mice (Fig. 2I and M).

These data indicate that SOD3 overexpression in CAFs promotes tumor invasion into the blood and lymphatic vessels.

### SOD3 overexpression in CAF promotes tumor metastasis to lymph nodes and the epithelial-mesenchymal transition process in LUAD

**Fig. 3:**
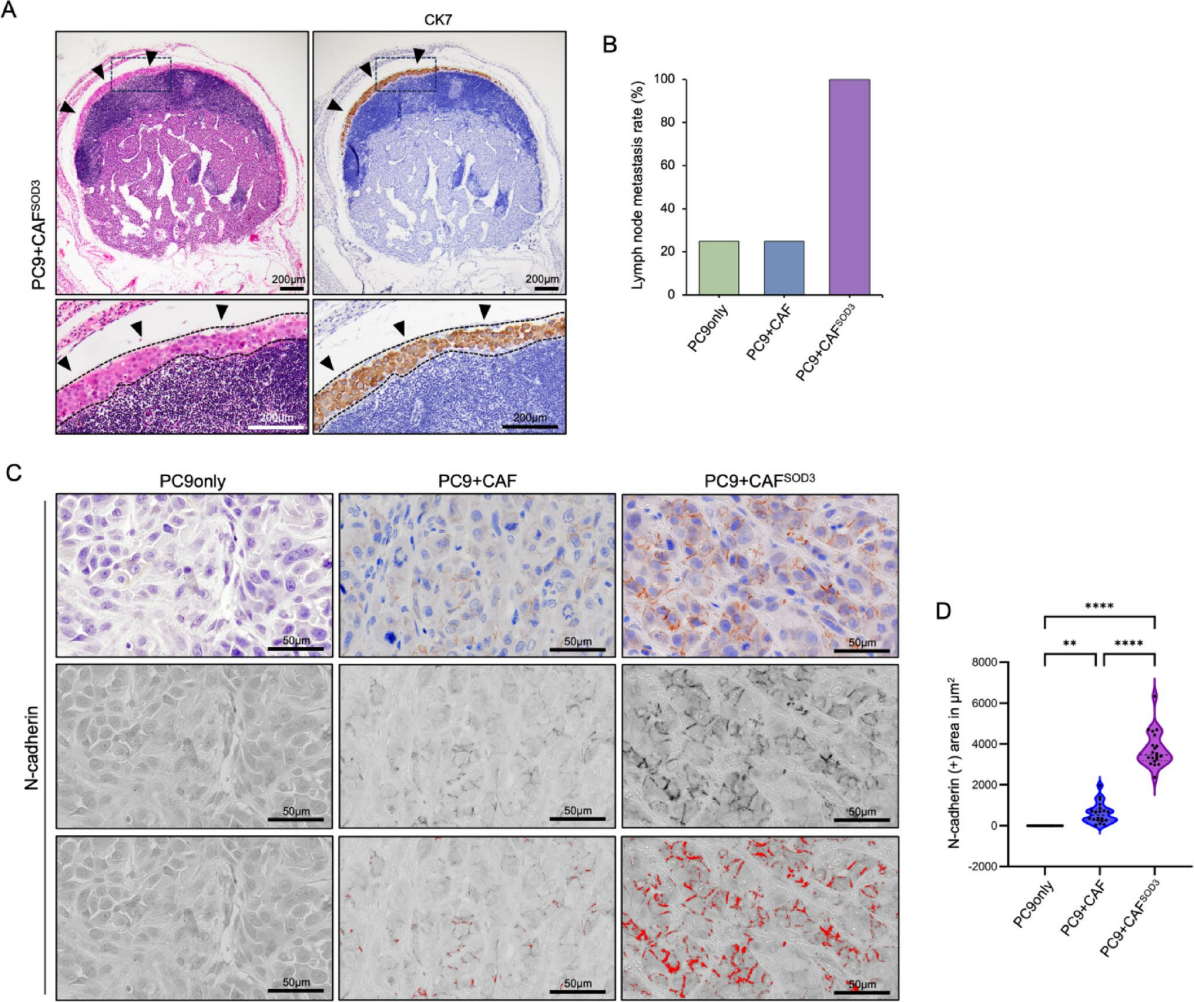
SOD3 overexpression in CAF enhances the lymph node metastasis and EMT in PC9 cells. **A** Representative image of the metastatic lymph node in PC9 + CAF^SOD3^ xenograft model. Metastatic tumor cells were detected in the lymph node by CK7 IHC. The dotted line outlines the metastatic tumor region. Arrowheads indicate the CK7-positive tumor cells within the lymph node. **B** Quantification of lymph node metastasis rates among different groups. **C** Representative IHC images of N-cadherin in tumor samples. Upper panel, IHC images of N-cadherin. Middle panel, signal detection by ImageJ. Lower panel, Quantification of N-cadherin expression using ImageJ (red color, N-cadherin signal). **D** Quantification of N-cadherin signal area. Data were presented as the area (µm^2^) per image. CAF, cancer-associated fibroblast extracted from a LUAD patient; CAF^SOD3^, SOD3 overexpression in CAF. PC9 only, PC9 only implanted xenograft; PC9 + CAF, xenograft model with PC9 and CAF; PC9 + CAF^SOD3^, xenograft model with PC9 and CAF^SOD3^. *n* = 4, five images with high-magnification per sample. Statistical analyses were performed using one-way ANOVA followed by Tukey’s multiple-comparison post hoc test; **P < 0.01, ****P < 0.0001

Given the observed lymphatic vessel invasion of the tumor, we next investigated lymph node metastasis in tumor-transplanted mice. Lymph nodes were harvested, formalin-fixed, and embedded in paraffin for histological analysis. HE staining of lymph node sections revealed tumor cell invasion (Fig. 3A). To examine the presence of tumor cells, we performed IHC staining with an anti-human cytokeratin7 (CK7) antibody, a specific marker for human-derived cells. CK7 staining further confirmed the tumor cell invasion in the lymph nodes (Fig. 3A). Among the groups, the metastasis rate was significantly higher in the mice group transplanted with PC9 and CAF^SOD3^ (100%) than that transplanted with PC9 and non-overexpressed CAFs (25%) and that transplanted with PC9 without CAFs (25%) (Fig. 3B, Supplementary Fig. 3A).

Again, to investigate the role of CAF-derived SOD3 in the epithelial-mesenchymal transition (EMT) of PC9 cells, we prepared CM from cultured CAFs with or without SOD3 overexpression (Supplementary Fig. 3B) and stimulated PC9 with CM for 48 h. Western blotting confirmed the secretion of SOD3 into the conditioned medium (CM) by CAF^SOD3^ (Supplementary Fig. 3C). To examine its functional impact, we treated PC9 cells with CM for 48 h and evaluated EMT marker expression. Specifically, we assessed E-cadherin as an epithelial marker and N-cadherin as a mesenchymal marker. The results showed that E-cadherin was downregulated, while N-cadherin expression remained unchanged in PC9 cells treated with CAF^SOD3^-CM (Supplementary Fig. 3D). These findings suggest the induction of a partial EMT phenotype. We propose that this reflects an early or context-dependent EMT response mediated by SOD3. Consistent with the in vitro observations, we confirmed that tumor cells of the SOD3-overexpressed CAF co-transplantation group exhibited a higher expression of N-cadherin than the other groups (Fig. 3C and D). Taken together, these findings indicate that CAF-derived SOD3 promotes EMT in tumor cells.

Again, to investigate the autocrine role of SOD3 in the EMT process, we established a SOD3 overexpression cell line of PC9 cells (PC9^SOD3^) using a human-SOD3 plasmid, which expresses mCherry (Supplementary Fig. 3E). SOD3 overexpression led to downregulation of E-cadherin and upregulation of N-cadherin, indicating that SOD3 promotes EMT through an autocrine mechanism (Supplementary Fig. 3F). In addition, PC9^SOD3^ exhibited increased intracellular ROS activity (Supplementary Fig. 3G), suggesting that SOD3-mediated ROS regulation may contribute to EMT induction.

### SOD3 overexpression in LUAD patient-derived CAFs affects the enrichment of TME shaping gene sets

**Fig. 4:**
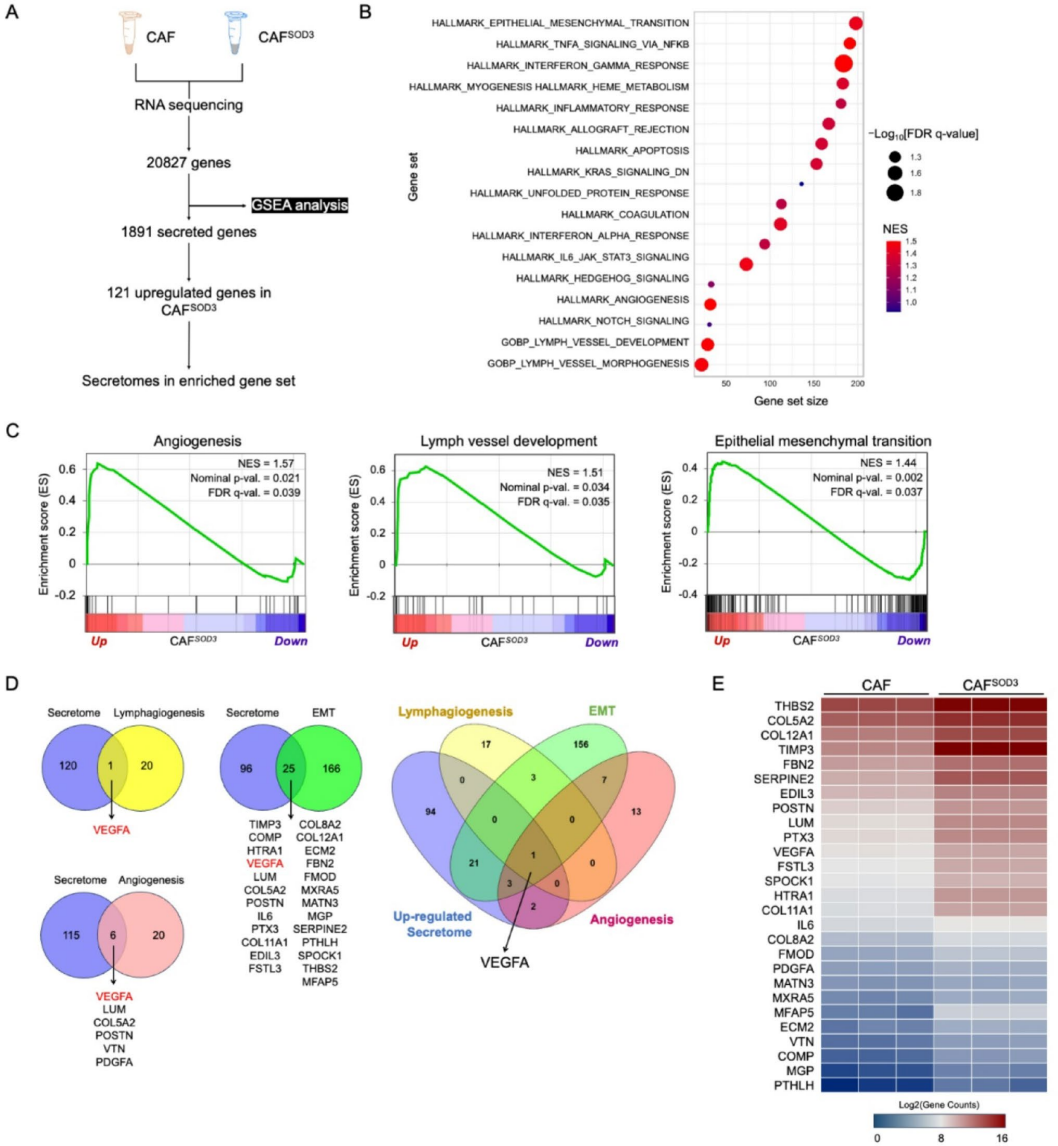
RNA-sequencing analysis of SOD3-overexpressed CAF reveals upregulation of genes related to angiogenesis, lymphangiogenesis, and EMT. **A** Schematic overview of RNA-seq analysis on SOD3-overexpressed CAF. GSEA was performed on 2082 genes, identifying overlapping genes with the secretome-related pathway. **B** GSEA results indicate that gene sets related to angiogenesis, lymphangiogenesis, and EMT were significantly up-regulated in CAF^SOD3^. **C** Enrichment plots of angiogenesis, lymph vessel development, and EMT. **D** Venn diagram generated using Venny 2.1, illustrating overlapping genes among enriched gene sets and secretomes. **E** Heatmap presentation of the overlapped gene counts. Color presentation range represents the Log2(Gene counts). CAF, cancer-associated fibroblast extracted from a LUAD patient. CAF^SOD3^, SOD3 overexpression in CAF

To gain mechanistic insight into the role of SOD3 secreted from CAFs in TME, we performed RNA sequence analysis on CAF^SOD3^ and compared it to CAF. As shown in Fig. 4A, gene set enrichment analysis was conducted on a total of 20827 genes. Given that CAF conveys its effects to neighboring cells in TME through its secretory factors, we analyzed the secretomes of CAF and CAF^SOD3^. Here, we identified 1891 secreted genes, from which we selected 121 up-regulated secretomes and identified overlapping genes with enriched genes in CAF^SOD3^.

Our analysis revealed that CAF^SOD3^ exhibited enrichment in extracellular matrix regulation genes, including angiogenesis, lymphatic vessel development, and EMT (Fig. 4B and C). Further analysis showed that 6, 1, and 25 enriched genes in angiogenesis, lymphangiogenesis, and EMT, respectively, overlapped with up-regulated secretomes (Fig. 4D and E). Notably, vascular endothelial growth factor A (VEGFA) was a common secretome shared across all enriched gene sets, suggesting that CAF-derived SOD3 mediates VEGFA-dependent angiogenesis, lymphangiogenesis, and EMT process in LUAD. We also confirmed the upregulation of VEGFA mRNA and protein level in CAF^SOD3^ compared to CAF and CAF^mCherry^ (Supplementary Fig. 4A, and 4B).

To validate the downstream signalling of VEGFA secreted from CAF^SOD3^, we further investigated whether CAF^SOD3^ could enhance VEGFR2 phosphorylation in HUVECs. HUVECs were stimulated with CM from CAF, CAF^mCherry^, and CAF^SOD3^. Endothelial growth medium with or without VEGF (50 ng/ml) was used as a positive or negative control, respectively. The results showed that CAF^SOD3^-CM induced a greater level of VEGFR2 phosphorylation compared to CAF-CM and CAF^mCherry^-CM, suggesting that SOD3 overexpression in CAF promotes the VEGF signaling pathway through VEGFA (Supplementary Fig. 4C). In addition, to evaluate the SOD3 effect on angiogenesis and lymphangiogenesis, HUVEC tube formation and migration assays were performed under treatment with the respective CM. CAF^SOD3^-CM significantly enhanced tube formation and migration in HUVECs (Supplementary Fig. 4D–G), supporting the role of SOD3 in promoting angiogenic and lymphangiogenic activity.

**Fig. 5:**
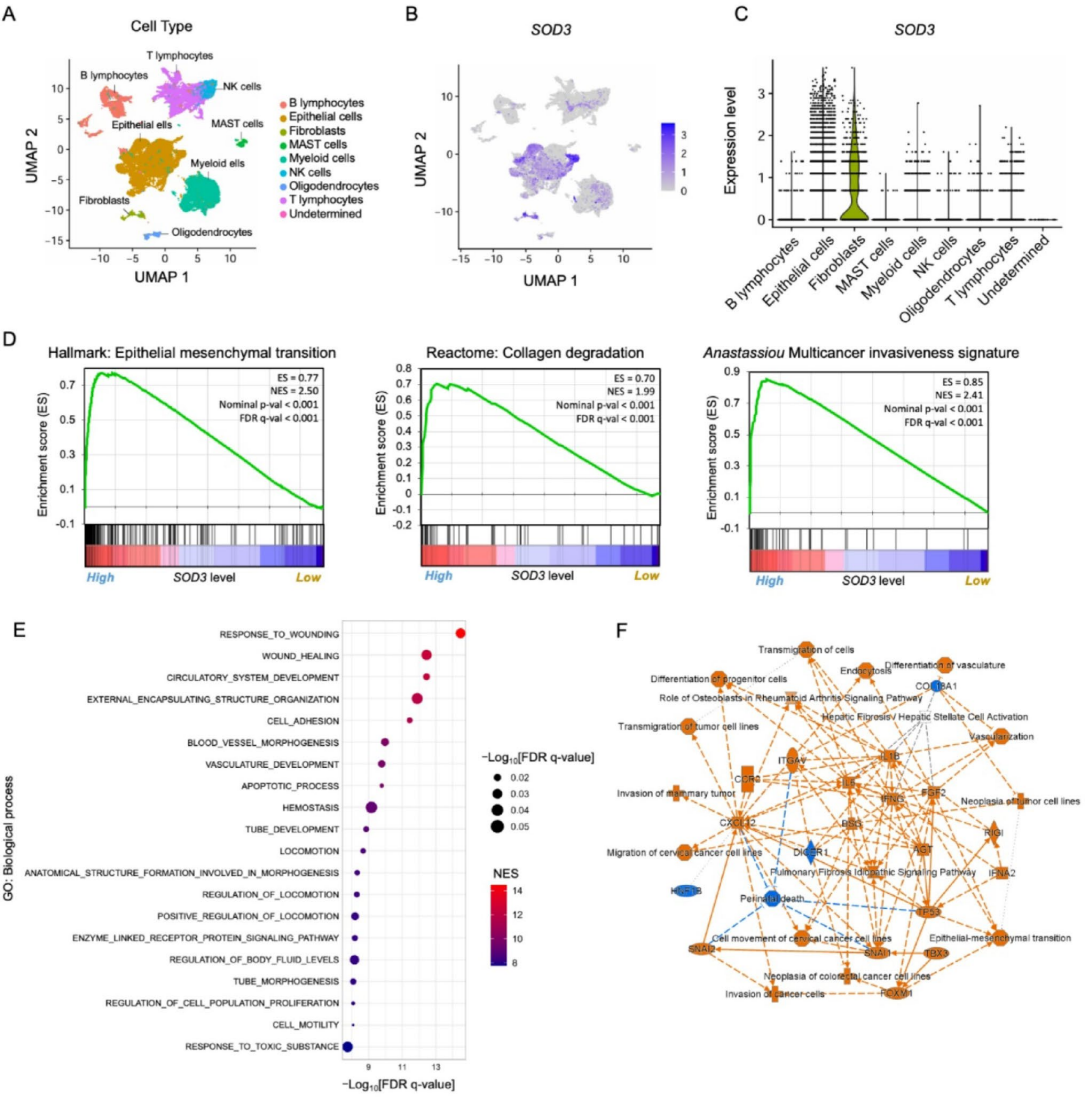
Single-cell RNA sequence analysis of advanced stages of LUAD clinical samples reveals SOD3 high expression in fibroblasts, which positively correlates with the cancer exacerbation features. **A** A UMAP plot of advanced stages of LUAD clinical samples, color-coded by major cell lineages. **B** UMAP plot of the SOD3 gene expression across major cell types. **C** Violin plot indicating SOD3 expression level in major cell types. **D** Gene sets responsible for cancer exacerbation features: EMT, collagen degradation, and multicancer invasiveness signature, positively correlated with SOD3 high expression in fibroblasts. **E** Bubble plot showing the up-regulated biological processes in SOD3 high fibroblasts of advanced-stage LUAD patients. The top 20 enriched GO terms highlight ECM remodeling and vascular development. **F** The IPA graphical summary predicted activation of tumor-promoting processes, such as EMT, vasculogenesis, and ECM remodeling, in SOD3-high fibroblasts from advanced-stage LUAD

Furthermore, sc RNA sequence analysis revealed that in advanced-stage tumor groups, functional analysis of SOD3-expressing fibroblasts observed the enrichment in gene sets associated with the EMT process, collagen degradation, and multi-cancer invasiveness signature (Fig. 5A-D). Gene ontology (Biological Process) analysis of genes up-regulated in SOD3-high fibroblasts compared to SOD3-low fibroblasts from advanced-stage LUAD highlighted enrichment in pathways related to ECM remodeling and vascular development, suggesting pro-tumorigenic traits associated with SOD3-rich fibroblasts (Fig. 5E). Additionally, IPA analysis predicted activation of tumor-promoting processes such as EMT, vasculogenesis, and ECM remodeling in SOD3-high fibroblasts from advanced-stage LUAD (Fig. 5F).

Taken together, these data indicate that SOD3 high expression in fibroblasts is associated with the pro-tumorigenic traits in LUAD.

### SOD3 expression in fibroblasts positively correlates with the rate of lymphatic invasion of tumors in LUAD clinical samples

**Fig. 6:**
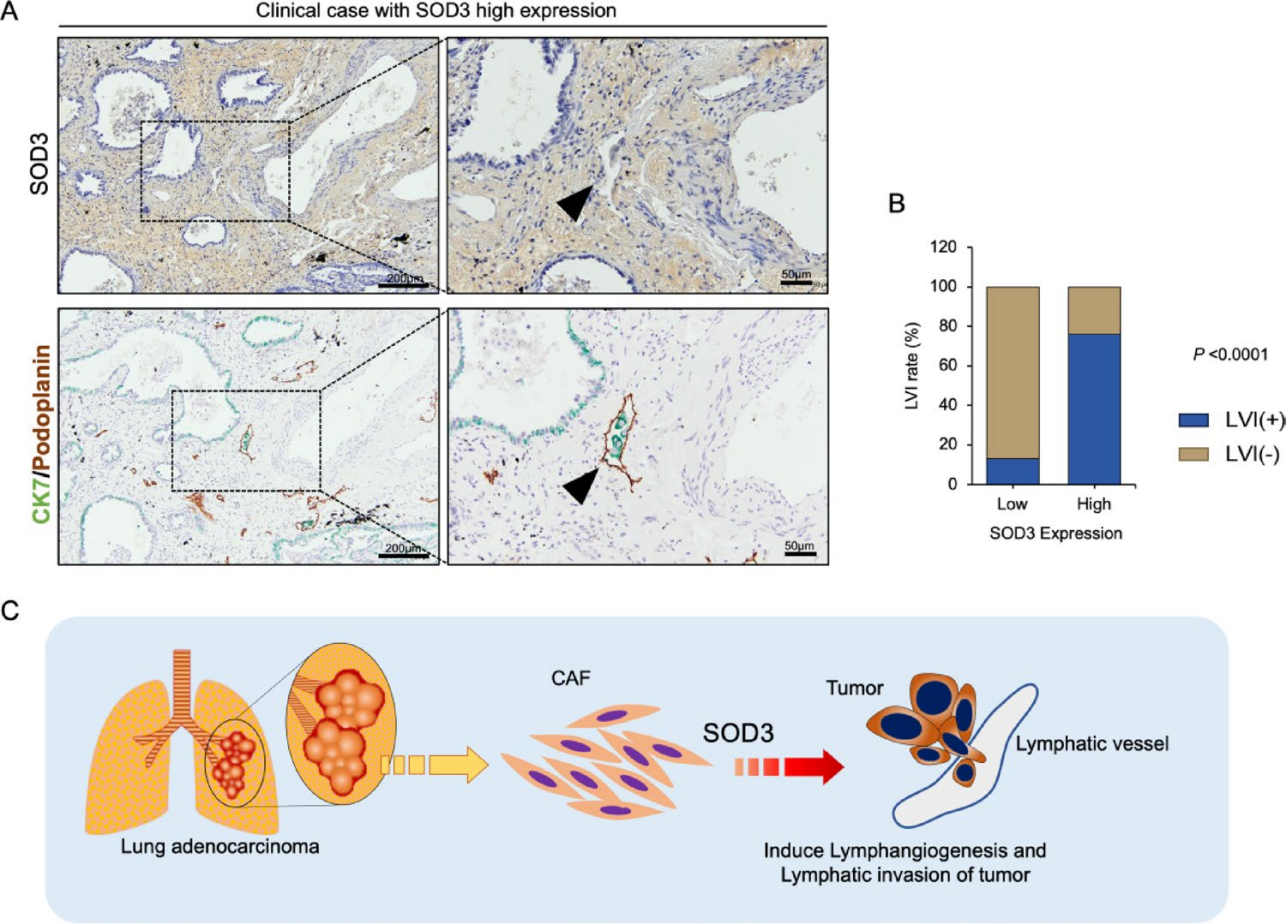
SOD3 expression in fibroblasts positively correlates with the rate of lymphatic invasion of tumors in LUAD patients. **A** Representative image of double staining for CK7 (green) and podoplanin (brown). Arrowheads show the tumor invasion into a podoplanin-positive lymph vessel. **B** Statistical analysis showing the correlation between lymphatic vessel invasion (LVI) and SOD3 expression in LUAD clinical samples. Statistical analysis was performed using Fisher’s exact test. *P* < 0.05 is considered significant. **C** Schematic diagram of the influence of CAF-derived SOD3 in the LUAD tumor microenvironment. High SOD3 expression in CAFs induces lymphangiogenesis and the lymphatic vessel invasion of the tumor in LUAD

Finally, we analyzed the correlation between tumor invasive character and SOD3 expression in the fibroblasts and ECM within the LUAD TME. LUAD patient-derived biopsies were carried out by the IHC on SOD3 and divided into two groups: low and high SOD3 expression. To analyze the tumor invasive feature, we performed the double staining of CK7 together with podoplanin, a lymphatic vessel marker. As shown in Fig. 6A, LUAD tumors with high SOD3 expression exhibited a higher incidence of intralymphatic tumor invasion. Additionally, comparing the different SOD3 expression levels, we observed a positive correlation between high SOD3 expression and an increased rate of lymphatic vessel invasion (Fig. 6B). Taken together, these results suggest that high SOD3 expression in CAF may enhance the tumor invasion into lymphatic vessels in LUAD and contribute to lymph node metastasis (Fig. 6C).

## Discussion

Imbalances in redox metabolism are common characteristics of human cancers [23]. ROS, such as O_2_^−^ and H_2_O_2_, are major byproducts of cancer cells and are increased in the tumor microenvironment [24]. Elevated ROS levels induce cytotoxic and mutagenic effects in cells, resulting in cell death, apoptosis, and senescence. Notably, at low levels, ROS plays critical roles in cellular signaling, regulating functions such as cell proliferation [25], migration [26], angiogenesis [27, 28] and lymphangiogenesis [29–31]. The increase in both intracellular and extracellular ROS levels is primarily due to decreased activity of antioxidant enzymes. Recent studies have focused on modulating redox metabolism by targeting antioxidant enzymes [23]. SOD3 is the only member of the SOD family present in the extracellular space, where it acts as a significant scavenger of ROS, thereby regulating redox homeostasis. SOD3 expression is highly cell-type specific and is abundantly present in the lung [14, 32–35]. In this study, we demonstrate the role of SOD3 in promoting lymphangiogenesis in LUAD.

SOD3 catalyzes the dismutation of O_2_^−^ into O_2_ and H_2_O_2_, protecting cells from oxidative stress. Although SOD3 is generally downregulated and considered a tumor suppressor gene in most cancers, its role appears to be dual and context-dependent. Two factors are crucial for understanding this duality: (1) the source of SOD3 within the heterogeneous tumor microenvironment and (2) the end product of the SOD3 reaction, specifically H_2_O_2_. In breast cancer, the relationship between SOD3 expression and disease progression is complex. Loss of SOD3, often due to promoter DNA hypermethylation, has been associated with poorer patient survival [36]. Conversely, certain studies report elevated SOD3 expression in highly metastatic breast cancer cell lines, where it may promote migratory behavior [37]. This paradox reflects the context-specific influence of SOD3, which may vary depending on cancer subtype, cellular compartment (tumor cells vs. stromal cells), and broader microenvironmental factors. In thyroid cancer, for instance, mesenchymal stem/stromal cells (MSCs) isolated from papillary thyroid carcinoma (PTC) exhibit higher SOD3 expression than MSCs from benign thyroid tissues. Interestingly, this stromal SOD3 promoted cancer cell proliferation but suppressed migration in co-culture experiments, suggesting a differential impact compared to tumor cell-intrinsic SOD3 [38]. Together, these findings underscore the importance of dissecting cell-type-specific roles of SOD3 in distinct tumor microenvironments to understand its implications in cancer biology.

Unlike previous studies, our research identifies CAFs as the primary source of SOD3 production in the LUAD tumor microenvironment. Through scRNA-seq and immunohistochemical analysis of clinical samples, we show that CAFs mainly produce SOD3 in the LUAD tumor microenvironment. CAFs play a pivotal role in facilitating tumor progression, migration, and metastasis by secreting various cytokines, chemokines, and growth factors. Importantly, SOD3 expression in stromal cells has been shown to modulate cancer cell growth and migration in thyroid cancer [38]. Here, we report that CAF-derived SOD3 promotes angiogenesis, lymphangiogenesis, and metastasis of LUAD. In addition, we report that high SOD3 expression in stroma are of LUAD clinical samples positively related to recurrence and intralymphatic tumor invasion. TCGA data analysis also provided that high expression of SOD3 in LUAD patients presents poorer overall survival status.

Angiogenesis and lymphangiogenesis provide critical pathways for metastatic cancer cells to disseminate to lymph nodes and distant organs [39]. While SOD3 is generally regarded as a tumor suppressor gene, its end product, H_2_O_2_, exhibits a dual nature. On one hand, H_2_O_2_ acts as an oxidative stress agent, which at high concentrations can have detrimental effects on cells, tissues, and the whole body. On the other hand, H_2_O_2_ serves as a second messenger in signaling pathways related to cell survival, growth, and proliferation [25]. This duality suggests that SOD3 might function as either a tumor facilitator or tumor suppressor, depending on the tumor microenvironment and its interactions during development. Under physiological conditions, endogenous H_2_O_2_ plays a critical role in regulating signaling pathways within vascular smooth muscle cells, affecting their proliferation, migration, differentiation, and phenotype. However, excessive levels of exogenous H_2_O_2_ can result in pathological conditions such as vascular inflammation and calcification [40]. In our study, SOD3 overexpression CAF produced a significant higher ROS activity than control CAFs. It is well-known that ROS (H_2_O_2_) stabilizes HIF-1α even under normoxic conditions, leading to increased VEGF transcription [41, 42]. Additionally, ROS can activate NF-kB promoting VEGF transcription [43]. Notably, SOD3-derived H_2_O_2_ enhances VEGF/VEGFR2 signaling, which promotes endothelial cell proliferation, migration, and angiogenesis [44]. Similarly, H_2_O_2_ promotes lymphatic endothelial cell proliferation by stimulating the VEGFC/VEGFR3 pathway [31, 45]. Further evidence highlights the interplay between SOD3 and VEGFC VEGF-C. In breast cancer models, restoring SOD3 under VEGFC knockdown conditions enhances primary tumor development and metastasis, indicating a synergistic relationship between VEGFC and SOD3 in tumor progression [46]. VEGFA, another key player, is well-established as a stimulator of lymphangiogenesis and blood vessel formation [47]. VEGFA has been shown to increase regional lymph node lymphangiogenesis, thereby facilitating tumor metastasis to the regional lymph nodes [48–50]. In alignment with these findings, our RNA sequence analysis of the secreted genes from CAF^SOD3^ revealed significant upregulation of VEGF, a gene prominently involved in lymphatic vessel development, angiogenesis, and metastasis. Moreover, CM from CAFSOD3 induced greater phosphorylation of VEGFR2 in HUVECs, suggesting activation of the VEGFA/VEGFR2 signaling pathway. Consistent with these findings, our in vivo studies also demonstrated that SOD3-rich CAFs promote angiogenesis, lymphangiogenesis, and lymph node metastasis of LUAD cancer cells.

Lymphatic endothelial cells in tumor-draining lymph nodes undergo reprogramming to acquire an immunosuppressive phenotype, thereby facilitating tumor metastasis [51, 52]. One key mechanism involves the expression of Programmed Death Ligand 1 (PD-L1) on lymphatic endothelial cells, which promotes a pro-tumor immune response. PD-L1 interacts with Programmed Cell Death Protein 1 (PD-1) on T cells, leading to T cell apoptosis and reduced anti-tumor immunity [53, 54]. A study by Cecilia Roux et al. demonstrates that increased ROS in macrophages, driven by impaired antioxidant mechanisms and chemotherapy, results in upregulation of PD-L1 expression. This process promotes VEGFA release, drives angiogenesis, and suppresses T cell-mediated anti-tumor responses. This study also highlighted the potential benefits of combining PD-L1 blockade with chemotherapy for improved therapeutic outcomes [55]. Furthermore, bioinformatic analyses of lung cancer patient datasets from the Gene Expression Comprehensive Database (GEO) and the Cancer Genome Atlas (TCGA) by Yundi Zhang et al. revealed a correlation between SOD3 expression and immune checkpoint molecules, such as PD-1 and Cytotoxic T-lymphocyte-associated protein 4 (CTLA-4) [19]. Other reports have indicated that high SOD3 expression in endothelial cells enhances laminin α4 production, which selectively increases T-cell infiltration into the tumor [56]. Additionally, restoring SOD3 in tumor endothelial cells has been shown to normalize tumor vasculature and improve tissue perfusion, thereby enhancing the efficacy of doxorubicin in lung cancer mouse models [18]. These findings suggest that SOD3 expression could serve as a biomarker for tailoring PD-L1 targeted therapies or for designing combination therapies that simultaneously target PD-L1 and SOD3.

This study has several limitations. While we used patient-derived human CAFs and confirmed key findings in clinical LUAD samples, the in vivo experiments were conducted in xenograft models, where human tumor cells and CAFs interact with murine stromal components. Such cross-species interactions may not fully recapitulate the complexity of the human tumor microenvironment, particularly in terms of endothelial cell signaling, matrix composition, and cytokine responses. Additionally, the use of immunodeficient mice precludes the involvement of the immune system, which is known to modulate tumor progression and stromal dynamics. Another limitation is the absence of a definitive loss-of-function model for SOD3. Although we attempted CRISPR/Cas9-mediated knockout of SOD3 in CAFs, complete depletion resulted in significant cell death and reduced viability, indicating that SOD3 is essential for CAF survival under our experimental conditions. Consequently, we utilized control CAFs with low endogenous SOD3 expression as comparators in our overexpression studies. While this approach allowed us to examine the functional impact of elevated SOD3 levels without inducing cytotoxic stress, it does not fully recapitulate the effects of SOD3 loss. Despite these limitations, the consistency between xenograft findings and clinical validation supports the relevance of our conclusions. Furthermore, while the precise mechanism underlying the interplay between SOD3 and lymph node metastasis was not directly addressed in this study, previous research, consistent with our RNA sequencing data, suggests that SOD3 mediates VEGF-dependent tumor progression, lymphangiogenesis, and metastasis in breast cancer [46]. These findings align with our observations, emphasizing SOD3’s role in exacerbating tumor progression.

Taken together, targeting cell-specific SOD3 may represent a promising treatment strategy for cancer. Our findings further propose that SOD3-rich CAFs may serve as a prognostic biomarker and a therapeutic target for LUAD patients. However, therapeutic targeting of SOD3-rich CAF in the tumor microenvironment has significant challenges. One major obstacle is the stromal heterogeneity. Even the CAF population has diverse subpopulations with distinct functions, which have opposite effects. Thus, targeting the whole population may result in an unwanted outcome. Moreover, there are potential compensatory mechanisms among redox-regulating enzymes, and the physiological role of SOD3 in maintaining normal tissue homeostasis could limit the safety and specificity of such interventions or may result in off-target effects. Future studies are needed to develop strategies that selectively inhibit tumor-promoting SOD3 activity without disrupting its systemic protective functions.

## Electronic supplementary material

Below is the link to the electronic supplementary material.

Supplementary file 1 (DOCX 10 kb)

## Data Availability

No datasets were generated or analysed during the current study.

## Abbreviations

CAF: Cancer-associated fibroblasts
CAF^SOD3^: SOD3-overexpressing CAF
CAF^mCherry^: Empty vector–only transfected CAF
SOD3: Superoxide dismutase 3
LUAD: Lung adenocarcinoma
LYVE1: Lymphatic vessel endothelial hyaluronan receptor 1
LVI: Lymphatic vessel invasion
CK7: Cytokeratin 7
VEGFA: Vascular endothelial growth factor A
VEGFR2: Vascular endothelial growth factor receptor 2
*p*-VEGFR2: Phosphorylated vascular endothelial growth factor receptor 2
HUVECs: Human umbilical vein endothelial cells
α-SMA: Alpha smooth muscle actin
PDPN: Podoplanin
