# Supplementary material for: Cancer-associated fibroblast-derived SOD3 enhances lymphangiogenesis to drive metastasis in lung adenocarcinoma
Source: Angiogenesis. 2025 Sep 30;28(4):51. doi: 10.1007/s10456-025-10005-9 (PMC12484251; doi:10.1007/s10456-025-10005-9)
Supplement: Supplementary file 1 — (DOCX 10 kb) [file 10456_2025_10005_MOESM1_ESM.docx]

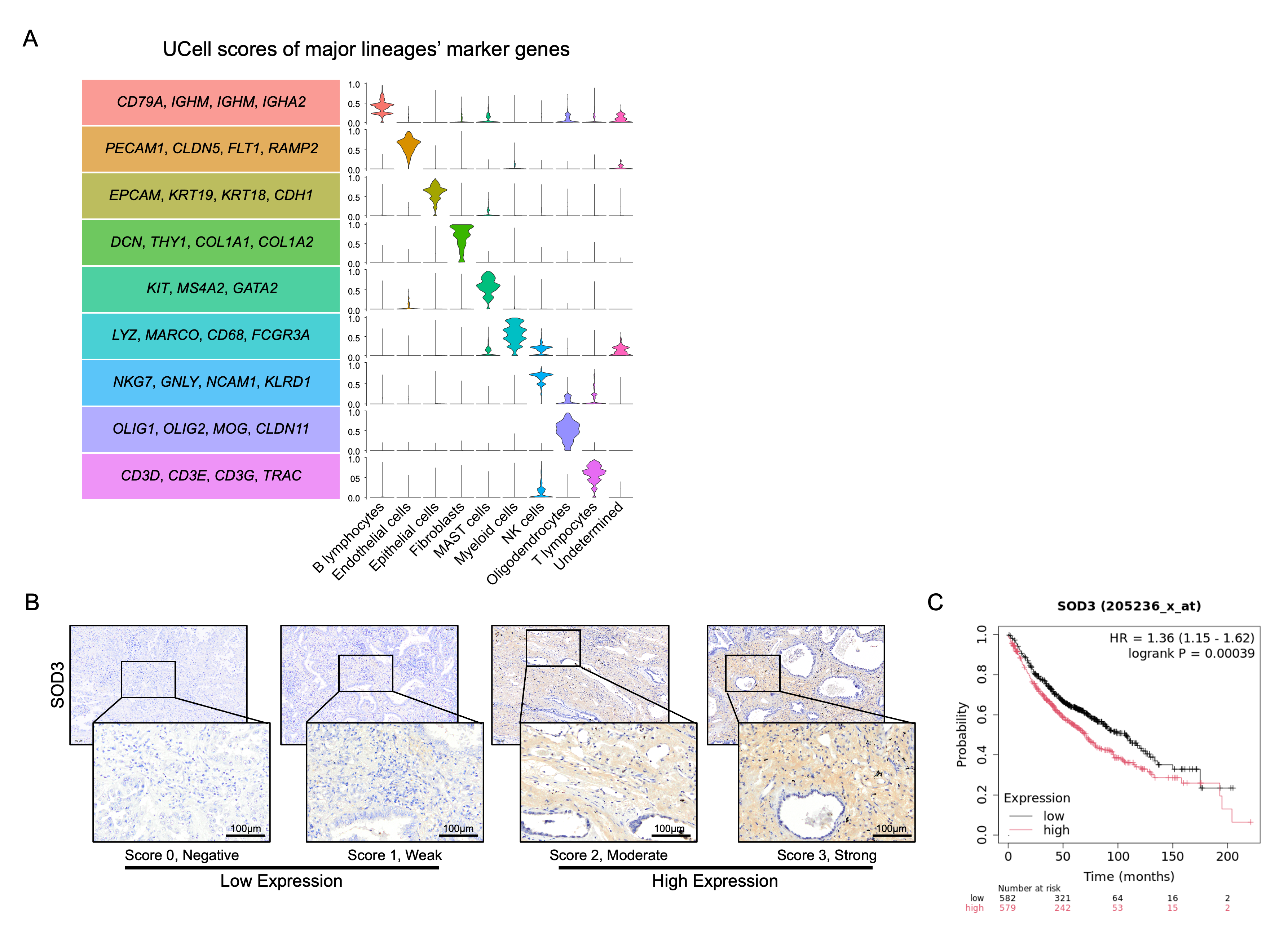


**Supplementary Fig. 1: High expression of SOD3 in LUAD patients showed a poor survival rate. (A)** Evaluation of cell-type annotation using the UCell module scores. Module scores were calculated using the UCell package based on marker genes reported in the original study (GSE131907). Violin plots display the distribution of module scores across clusters, showing specific enrichment in the corresponding annotated cell types. These results support the validity of the original cell-type assignments. **(B)** IHC scoring for SOD3 expression evaluation in LUAD clinical samples. SOD3 expression was defined from score 0 to 3 according to the expression intensity; score 0, Negative staining. 1, Weak staining. 2, Moderate staining. 3, Strong staining. Scores 0 and 1 are defined as Low Expression, and scores 2 and 3 are defined as High Expression. **(C)** Kaplan–Meier survival curve showing the overall survival of LUAD patients (TCGA dataset) stratified by SOD3 expression levels using the Kaplan–Meier Plotter tool. Patients were divided into high and low expression groups based on the median. Hazard ratio (HR), 95% confidence interval (CI), and p value from the log-rank test are shown.


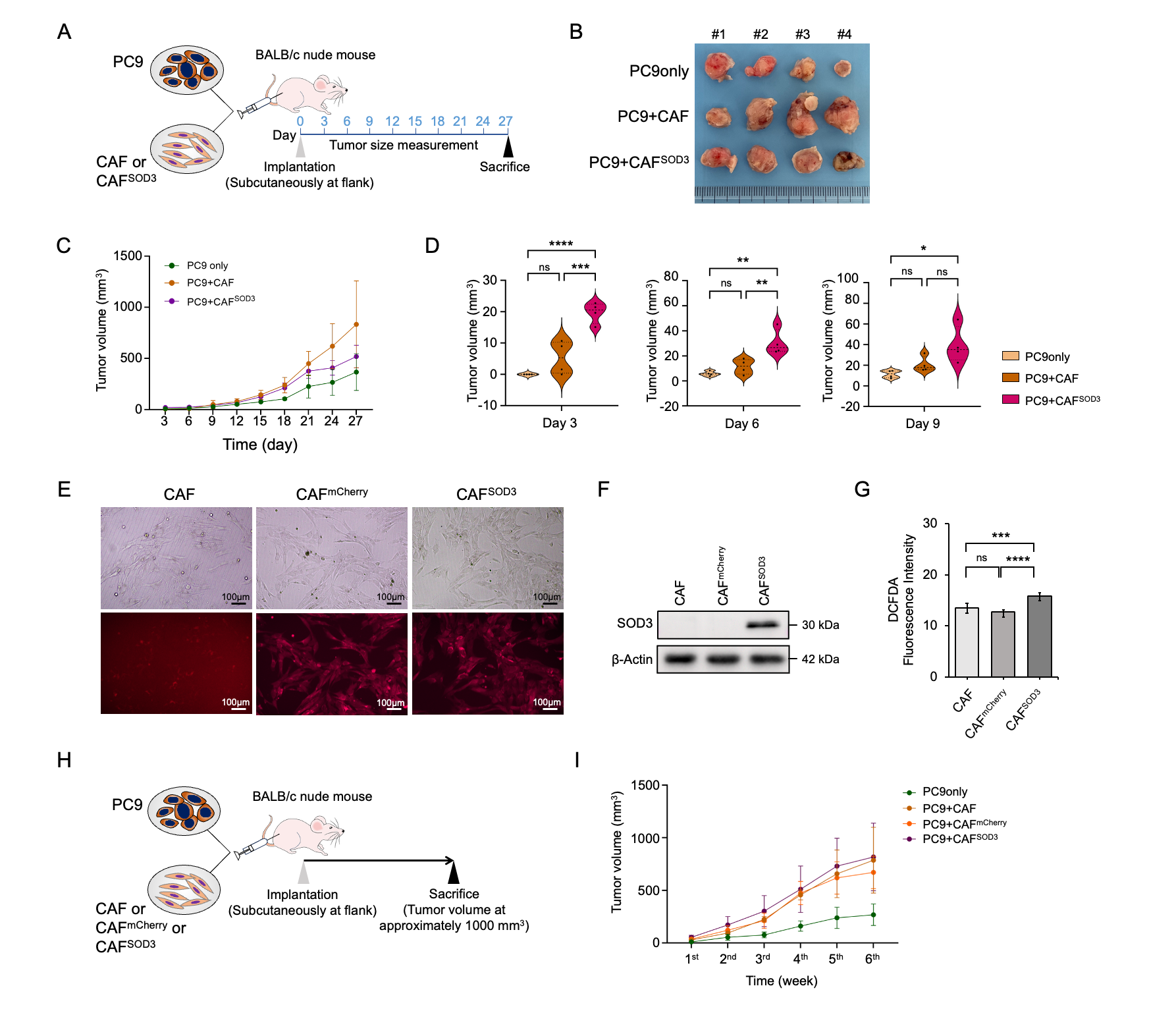


**Supplementary Fig. 2: LUAD xenograft with SOD3-overexpressed CAFs shows a tendency for increased tumor volume but no significant difference. (A)** Schematic timeline of tumor volume measurement in the LUAD xenograft model. Tumor volume was measured every three days, and mice were euthanized after 4 weeks of tumor implantation. **(B)** Representative images of harvested tumor samples from xenograft models. **(C)** Tumor volume was calculated using the formula of ½ (length × width^²^). Data was presented as mm^3^. **(D)** Quantification of tumor volume measurement at Day 3, 6, and 9. **(E)** Representative image of CAF, CAF^mCherry^, and CAF^SOD3^. Transfection was confirmed by mCherry expression under a fluorescent microscope. **(F)** Confirmation of SOD3 protein expression in established CAF^SOD3^ by immunoblot. **(G)** Measurement of intracellular reactive oxygen species (ROS) levels using the DCFDA (2′,7′-dichlorofluorescin diacetate) assay. Cells were incubated with DCFDA, and a plate reader measured fluorescence intensity. Data are presented as DCFDA fluorescence intensity. **(H)** Schematic diagram of tumor volume measurement in the LUAD xenograft model. Tumor volume was monitored weekly, and mice were euthanized when tumors reached approximately 1000 mm³. **(I)** Tumor volume was calculated using the formula: ½ (length × width²). No significant difference in tumor volume was observed between the two control groups (PC9 + CAF and PC9 + CAF^mCherry^) during the experimental period. Data was presented as mm^3^. CAF, cancer-associated fibroblast extracted from a LUAD patient; CAF^mCherry^, vector-only transfection in CAF; CAF^SOD3^, SOD3 overexpression in CAF; PC9 only, PC9 only implanted xenograft; PC9 + CAF, xenograft model with PC9 and CAF; PC9 + CAF^mCherry^, xenograft model with PC9 and CAF^mCherry^; PC9 + CAF^SOD3^, xenograft model with PC9 and CAF^SOD3^. n = 4. Statistical analyses were performed using one-way ANOVA followed by Tukey’s multiple-comparison post hoc test; ns = no significance, **P* < 0.05, ***P* < 0.01, ****P* < 0.001, *****P* < 0.0001.


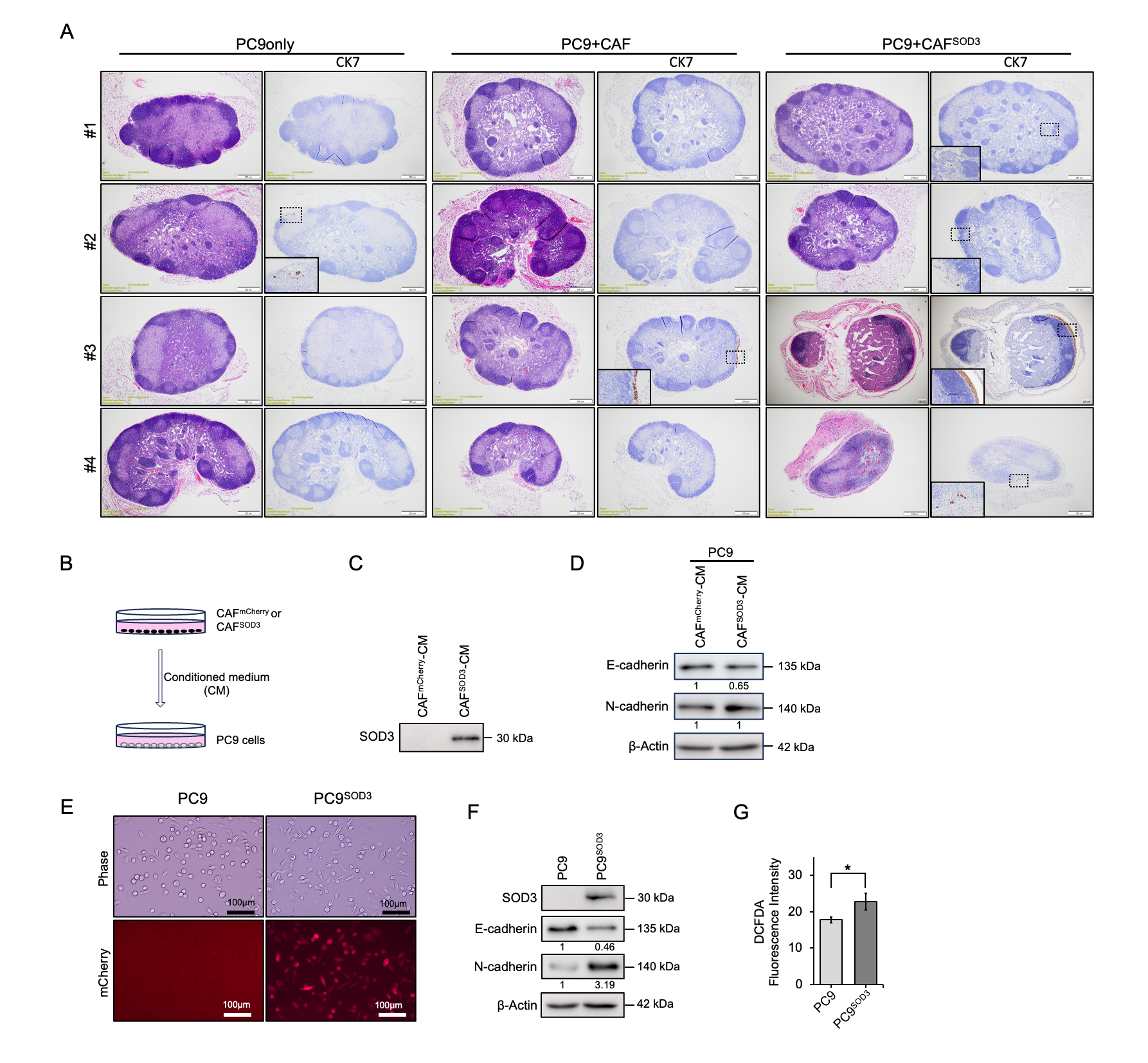


**Supplementary Fig. 3: SOD3-rich CAFs enhance lymph node metastasis and SOD3 functions as not only a paracrine effect (CAF to PC9) but also an autocrine effect in the LUAD cancer cell (PC9). (A)** Representative HE-stained images of lymph nodes from xenograft models. IHC for CK7 in lymph nodes is performed to detect metastatic tumor cells. Images in the islets represent the high magnification of CK7-positive cells. **(B)** Schematic diagram illustrating the collection of conditioned medium (CM) and subsequent stimulation of PC9 cells with CM. **(C)** Western blot analysis of SOD3 in CM collected from CAF, CAF^mCherry^, and CAF^SOD3^. SOD3 is secreted into the CM. **(D)** Western blotting on E-cadherin and N-cadherin in the PC9 cells treated with different CM to assess EMT marker expression. Normalized protein expression with β-Actin values is indicated below the corresponding protein bands. **(E)** Representative images of PC9, PC9^SOD3^. Overexpression was confirmed by mCherry expression under a fluorescent microscope. **(F)** Confirmation of SOD3 protein expression in established PC9^SOD3^ by immunoblot. Normalized protein expression with β-Actin values is indicated below the corresponding protein bands. **(G)** Measurement of intracellular reactive oxygen species (ROS) levels using the DCFDA (2′,7′-dichlorofluorescin diacetate) assay. Cells were incubated with DCFDA, and a plate reader measured fluorescence intensity. Data are presented as DCFDA fluorescence intensity. CAF, cancer-associated fibroblast extracted from LUAD patient; CAF^mCherry^, vector-only transfection in CAF; CAF^SOD3^, SOD3 overexpression in CAF; PC9 only, PC9 only implanted xenograft; PC9 + CAF, xenograft model with PC9 and CAF; PC9 + CAF^SOD3^, xenograft model with PC9 and CAF^SOD3^. n = 4. CM, conditioned medium; PC9^SOD3^, SOD3 overexpression in PC9 cells. Statistical analysis was performed using the Student’s t-test; **P* < 0.05.


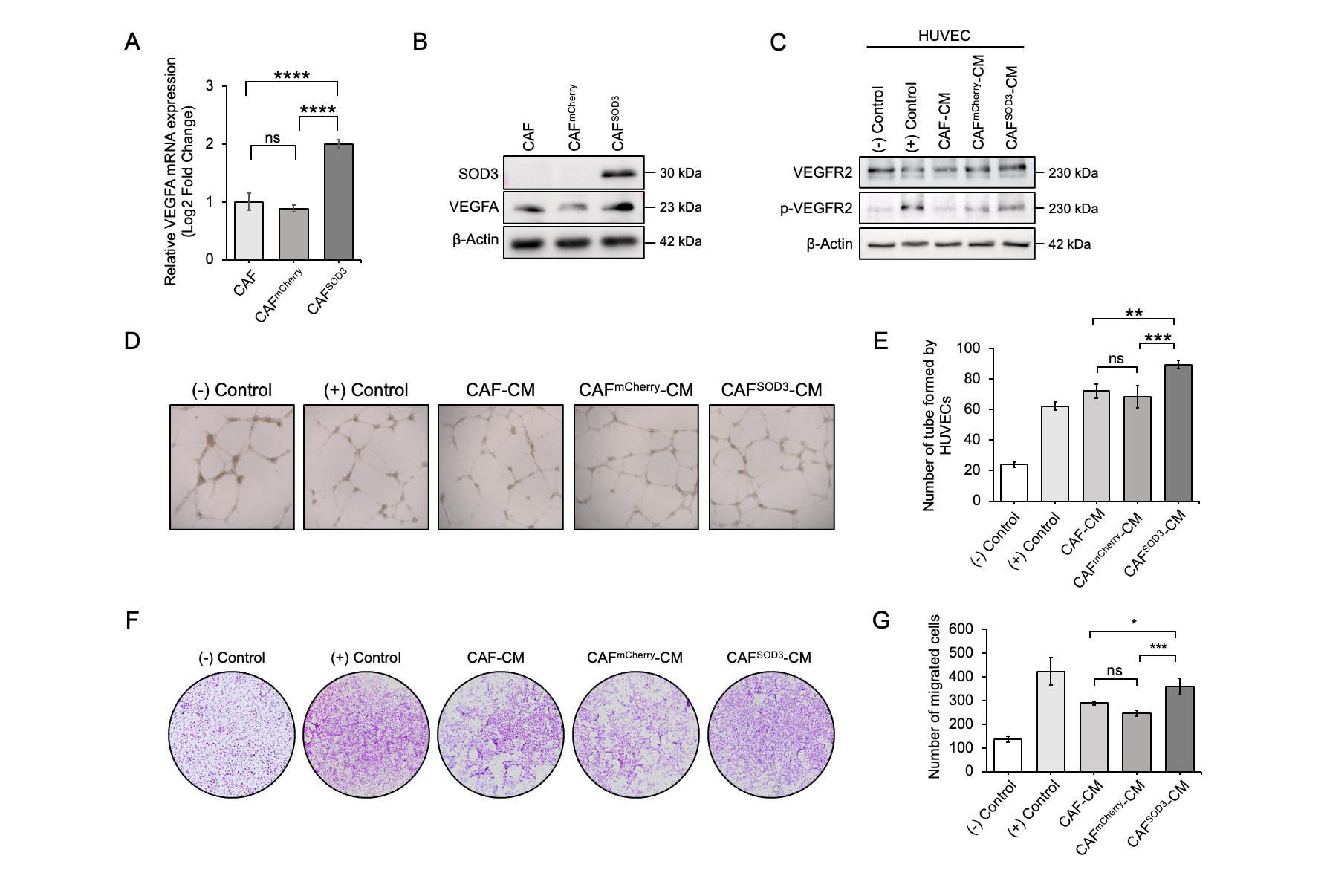


**Supplementary Fig. 4: SOD3 overexpression in CAF induces VEGFR2 phosphorylation in HUVEC, tube formation, and increases HUVEC cell migration. (A)** Quantitative RT-PCR of VEGFA mRNA expression in CAF, CAF^mCherry^, and CAF^SOD3^. CAF^SOD3^ showed significantly increased VEGFA expression compared to CAF and CAF^mCherry^. (B) Western blot analysis of VEGFA protein levels in CAF, CAF^mCherry^, and CAF^SOD3^, confirming upregulation of VEGFA in CAF^SOD3^. (C) Western blot analysis of VEGFR2 phosphorylation in HUVECs treated with conditioned medium (CM) from CAF, CAF^mCherry^, and CAF^SOD3^. CAF^SOD3^ induced greater VEGFR2 phosphorylation. **(D)** Representative images of tube formation by HUVECs treated with CM from CAF, CAF^mCherry^, and CAF^SOD3^. **(E)** Quantification of tube formation. The data represent the number of tube-like structures formed by HUVECs. **(F)** Representative images of HUVECs migration in transwell assays under treatment with CM from CAF, CAF^mCherry^, and CAF^SOD3^. **(G)** Quantification of migrated HUVEC. Endothelial growth medium with or without VEGF (50 ng/mL) was used as a positive and negative control, respectively. CAF, cancer-associated fibroblast extracted from LUAD patient; CAF^mCherry^, vector-only transfection in CAF; CAF^SOD3^, SOD3 overexpression in CAF; CM, conditioned medium. Statistical analyses were performed using one-way ANOVA followed by Tukey’s multiple-comparison post hoc test; ns = no significance, **P* < 0.05, ***P* < 0.01, ****P* < 0.001.


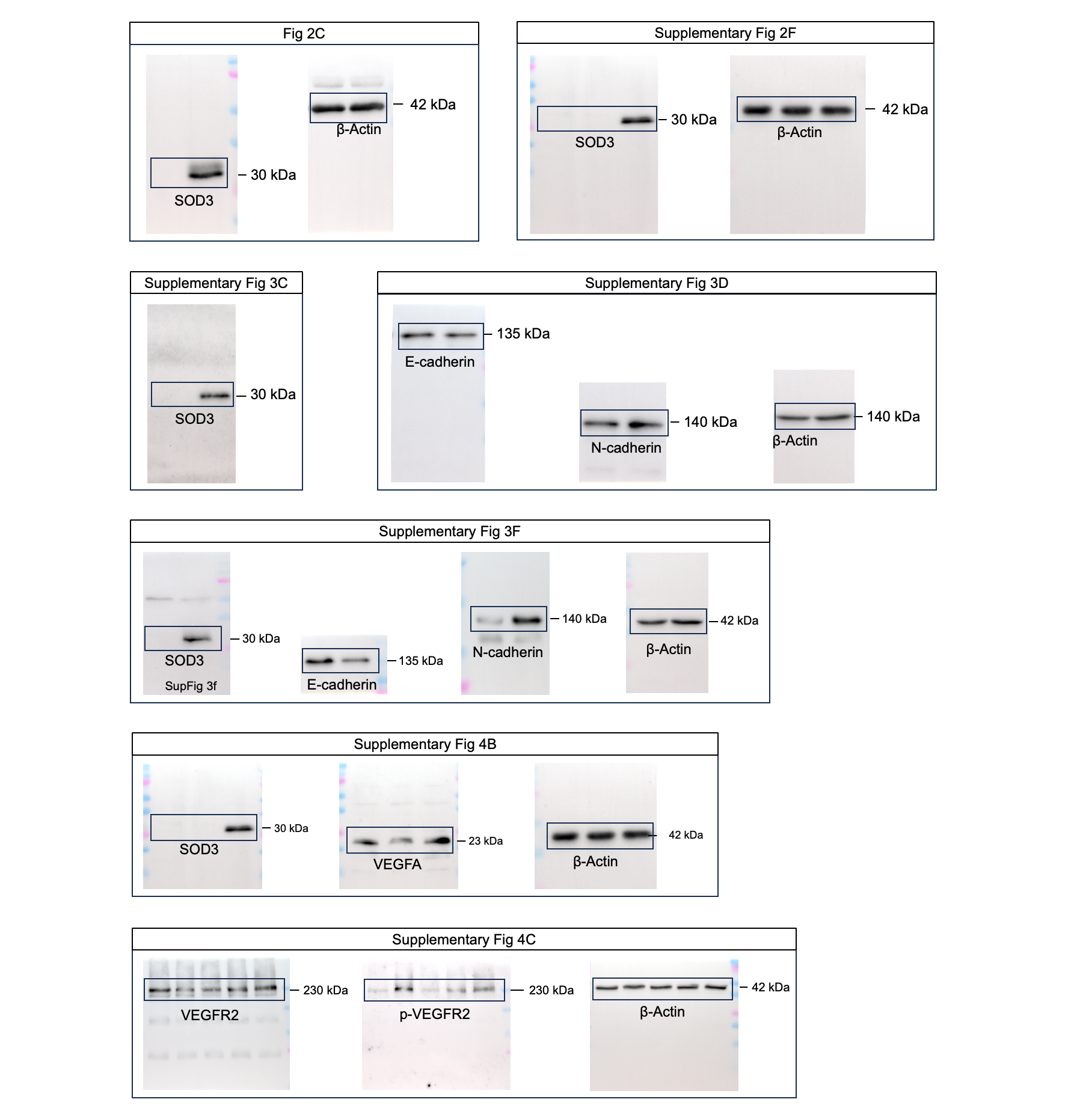


**Supplementary Fig. 5:** Full Western blots for key results. Full Western blots for all cropped images with the corresponding Figure and panel numbers are shown.

**Supplementary Table 1. Sequences for primers used to conduct conventional and qRT-PCR.**

| Gene | Primer | Sequence 5' to 3' |
| --- | --- | --- |
| VEGFA | Forward | AAGGGGCAAAAACGAAAGCG |
|  | Reverse | GCTCCAGGGCATTAGACAGC |
| β-Actin | Forward | CATGTACGTTGCTATCCAGGC |
|  | Reverse | CTCCTTAATGTCACGCACGAT |

Note: VEGFA, vascular endothelial growth factor A
